# Unravelling the sexual developmental biology of *Cystoisospora suis*, a model for comparative coccidian parasite studies

**DOI:** 10.3389/fcimb.2023.1271731

**Published:** 2023-10-25

**Authors:** Teresa Cruz-Bustos, Marlies Dolezal, Anna Sophia Feix, Bärbel Ruttkowski, Karin Hummel, Ebrahim Razzazi-Fazeli, Anja Joachim

**Affiliations:** ^1^ Department of Pathobiology, Institute of Parasitology, University of Veterinary Medicine Vienna, Vienna, Austria; ^2^ Platform for Bioinformatics and Biostatistics, Department of Biomedical Sciences, University of Veterinary Medicine Vienna, Vienna, Austria; ^3^ VetCore Facility (Proteomics), University of Veterinary Medicine Vienna, Vienna, Austria

**Keywords:** *Isospora suis*, coccidia, Apicomplexa, sexual development, proteome, metabolism, oocyst, gamonts

## Abstract

**Introduction:**

The apicomplexan parasite *Cystoisospora suis* has global significance as an enteropathogen of suckling piglets. Its intricate life cycle entails a transition from an asexual phase to sexual development, ultimately leading to the formation of transmissible oocysts.

**Methods:**

To advance our understanding of the parasite’s cellular development, we complemented previous transcriptome studies by delving into the proteome profiles at five distinct time points of *in vitro* cultivation through LC/MS-MS analysis.

**Results:**

A total of 1,324 proteins were identified in the *in vitro* developmental stages of *C. suis*, and 1,082 proteins were identified as significantly differentially expressed. Data are available via ProteomeXchange with identifier PXD045050. We performed BLAST, GO enrichment, and KEGG pathway analyses on the up- and downregulated proteins to elucidate correlated events in the *C. suis* life cycle. Our analyses revealed intriguing metabolic patterns in macromolecule metabolism, DNA- and RNA-related processes, proteins associated with sexual stages, and those involved in cell invasion, reflecting the adaptation of sexual stages to a nutrient-poor and potentially stressful extracellular environment, with a focus on enzymes involved in metabolism and energy production.

**Discussion:**

These findings have important implications for understanding the developmental biology of *C. suis* as well as other, related coccidian parasites, such as *Eimeria* spp. and *Toxoplasma gondii*. They also support the role of *C. suis* as a new model for the comparative biology of coccidian tissue cyst stages.

## 1 Introduction

Coccidian parasites are the largest group in the protozoan phylum Apicomplexa by number of species, comprising important pathogens of humans and animals worldwide. *Eimeria* spp. and *Cyclospora* spp. are the major genera of the family Eimeriidae, while *Toxoplasma gondii*, *Neospora* spp., *Sarcocystis* spp., *Cystoisospora* spp., *Hammondia* spp., and *Besnoitia* spp., among others, are closely related tissue cyst-forming parasites that belong to the family Sarcocystidae with monoxenous or (facultatively) heteroxenous life cycles (Votýpka et al., 2017). Among them, *T. gondii*, the causative agent of toxoplasmosis, is by far the best-studied species; however, it has a significant limitation as larger parts of its life cycle so far cannot be studied *in vitro* and animal models are limited (Warschkau and Seeber, 2023). *Cystoisospora* spp. infect various mammals, including dogs, cats, pigs, and primates (Lindsay and Blagburn, 1994). *Cystoisospora suis* is a frequent cause of diarrhoea and weight loss in neonatal piglets. Given the economic importance of the swine industry, molecular investigations on the development of *C. suis* are essential to elucidate new targets for disease control (Joachim and Shrestha, 2019). Like other coccidian parasites, *C. suis* has a complex life cycle that encompasses asexual and sexual reproduction (Lindsay and Current, 1984; Lindsay and Blagburn, 1994). Its endogenous development occurs exclusively in intestinal epithelial cells and culminates in the production of oocysts, which are adapted to survive under adverse climatic conditions after fecal excretion into the environment and subsequent sporulation. The complete development of *C. suis* was demonstrated *in vitro* by Lindsay and Current, 1984, but the authors failed to obtain viable, infectious oocysts (Feix et al., 2022a). Recently, the life cycle and sexual development of *C. suis* have been characterized in detail using an *in vitro* intestinal porcine epithelial cell (IPEC) culture system, providing a unique model for research on coccidian parasites (Worliczek et al., 2013; Feix et al., 2020; Feix et al., 2021; Cruz-Bustos et al., 2022; Feix et al., 2022a).

Asexual stages of Coccidia are obligately intracellular, and host cell invasion and intracellular persistence are well described in these stages (Mital and Ward, 2008; Frénal et al., 2017; Guérin et al., 2017). While all Coccidia also undergo sexual development, the majority of molecular characterization studies of this phase have been conducted on *T. gondii* and *Eimeria* spp. (Martorelli Di Genova and Knoll, 2020). Additionally, significant research has been carried out in *Hammondia*, *Besnoitia*, *Neospora*, and *Sarcocystis* (Cruz-Bustos et al., 2021; Ramakrishnan and Smith, 2021) as well as *C. suis* (Feix et al., 2020; Cruz-Bustos et al., 2022). However, understanding the metabolic requirements of the sexual stages is vital for targeted interventions at this life phase of the parasites (Ramakrishnan et al., 2019; Cruz-Bustos et al., 2022; Feix et al., 2022b), and so far, little is known about these due to often limited access to sexual stages of the mentioned parasites *in vitro*.

Recent genomic and proteomic studies have advanced our knowledge of apicomplexan metabolism (Wastling et al., 2009). These parasites depend on host nutrients, such as carbohydrates, amino acids, nucleosides, and vitamins, for their growth during asexual reproduction (Kloehn et al., 2021). Coccidian parasites harness central carbon metabolism pathways encompassing glucose uptake, glycolysis, gluconeogenesis, the TCA cycle, and the electron transport chain (Jacot et al., 2016). Environmental nutrient availability governs lipid scavenging, synthesis, and reassembly (Shunmugam et al., 2022). Recent investigations have unveiled the presence of a prokaryotic type II fatty acid (FASII) synthesis pathway in the apicoplast (Possenti et al., 2013). Purines and pyrimidines and their nucleotides are of fundamental importance in DNA replication and RNA transcription, as a source of cellular energy, and as cofactors or substrates in various cellular metabolic pathways (Uboldi et al., 2021). Folate, a key cofactor, mediates one-carbon metabolism, impacting amino acids, biosynthesis, epigenetics, and redox defence. *Toxoplasma gondii* acquires folate through both *de novo* synthesis and uptake from exogenous sources (Massimine et al., 2005). Apicomplexan parasites generally face the challenge of an oxidative environment within their host cells and meet this challenge with two classical antioxidant systems, the glutaredoxin and the thioredoxin systems, which provide thiol-disulphide pairs to regulate the redox balance (Bosch et al., 2015; Foyer et al., 2018). It is, however, not clear whether sexual stages display similar patterns of metabolism during their development.

Parasite stage transitions in Apicomplexan parasites require a precise regulation of gene expression. During the shift from asexual to sexual stages, this is primarily coordinated by epigenetic and transcriptional factors (Kim, 2018). The major transcription factors of Apicomplexa parasites are members of the ApiAP2 family, which have been found to activate or repress stage conversion (Painter et al., 2011). In more general terms, alteration of the chromatin structure is the most common form of epigenetic regulation (Jaenisch and Bird, 2003). The transition between different life cycle stages of *T. gondii* involves substantial changes in protein patterns and various levels of regulation, including essential posttranslational modifications such as phosphorylation and ubiquitination. Protein ubiquitination is crucial for protein turnover, cell signalling, and intracellular trafficking (Silmon De Monerri et al., 2015). The proteasome, a protease complex, is responsible for intracellular protein degradation and plays essential roles in cell cycle regulation, cell differentiation, and signal transduction pathways (Tanaka, 2009).

Advances in proteomic technologies have provided valuable insights into protein expression patterns in Apicomplexan parasites. Comparative proteomic analyses rely on sensitive mass spectrometry techniques, particularly shotgun approaches using liquid chromatography and mass spectrometry (LC-MS/MS). These methods provide a comprehensive analysis of the target proteome, enabling the identification and quantification of thousands of proteins in complex samples (Dailey, 2000; Huang et al., 2015; Monoyios et al., 2018). While LC-MS/MS has been successfully applied to proteome investigations on a limited set of developmental stages of *T. gondii* (Fritz et al., 2012a; Fritz et al., 2012b; Possenti et al., 2013; Wang et al., 2017; Garfoot et al., 2019) and *Eimeria* spp. (Lal et al., 2009), a comprehensive understanding of global protein expression patterns throughout the parasite development remains elusive. We here used LC-MS/MS to quantitatively analyse the *C. suis* proteome throughout the life cycle, from early multiplying merozoites to the immature and mature sexual stages and finally the unsporulated oocyst as the last stage of the endogenous development of *C. suis*. This comparative proteomics approach revealed distinct differences in the repertoires of metabolic enzymes expressed by different stages and provides important insights into the development and environmental adaptation of *C. suis* as a representative of the Coccidia.

## 2 Materials and methods

### 2.1 *Cystoisospora suis* oocyst collection

*Cystoisospora suis* oocysts (strain Wien I) were obtained from experimentally infected suckling piglets as described previously (Feix et al., 2020; Feix et al., 2021; Cruz-Bustos et al., 2022; Feix et al., 2022a). Pigs were kept in the animal facilities of the Institute of Parasitology, University of Veterinary Medicine Vienna, Austria.

### 2.2 Culture methods

Intestinal porcine epithelial cells (IPEC-1, ACC 705, Leibniz Institute DSMZ-German Collection of Microorganisms and Cell Cultures GmbH, Leibniz, Germany) were used as host cells *in vitro* and seeded in a density of 4 × 10^5^ cells per well (VWR, Vienna, Austria). Cells were grown under the conditions described in our previous works (Feix et al., 2020; Cruz-Bustos et al., 2022). After 24 h of cell growth, IPEC-1 cells were infected with 5 × 10^3^ sporozoites/well released from excysted oocysts and incubated further at 40°C under 5% CO_2_. The experiment employed a total of eight six-well plates, representing four biological replicates. Each biological replicate was composed of material pooled from two plates. We determined the growth differentiation of stages at each given time point by counting cells in a Neubauer chamber.

### 2.3 Experimental design, sampling, and protein extraction

For the sampling of sexual stages released from host cells, we collected cell culture supernatants every day, from day of cultivation (doc) 7 to day 14. The material was washed twice with phosphate-buffered saline (PBS; Gibco) and pelleted by centrifugation at 600 × *g* for 10 min. The numbers of merozoites, sexual stages, and oocysts were counted in a Neubauer-counting chamber for each given time point. For each day, four biological replicates were harvested and the mean numbers of each stage per biological replicate were calculated (**Figure S1**).

Pellets from the same wells were pooled to increase the number of parasites per sample, and the analysis was performed for five time points:

1. Day 7 (early merozoites; EMe) 
2. Day 8 (late merozoites; LMe) 
3. Day 10 (late merozoites and immature sexual stages; ISS)
4. Day 12 (mature sexual stages and unsporulated oocysts; MSS and UO)
5. Day14 (unsporulated oocysts)

To produce total protein lysates, frozen pellets were solubilized in lysis DIGE buffer (8 M urea, 4% (w/v) CHAPS, 40 mM TRIS [Tris(hydroxymethyl)aminomethane] base) containing DNase/RNase and protease inhibitors (Roche) and subjected to three rapid (2 min) cycles of freezing in liquid nitrogen/defrosting at 30°C with a vigorous vortex. Samples were centrifuged at 13,000 × *g* for 30 min at 4°C. The protein concentration of samples was determined by a Pierce 660 nm protein assay according to the manufacturer’s protocol (DS-11 FX+, DeNovix Inc., Wilmington, DE, USA). The workflow of the experiment is detailed in **Figure S2**.

### 2.4 Sample preparation for mass spectrometry

30 µg of the protein was filled up to 500 µl with 8 M urea in 50 mM TRIS and was loaded on to a 10-kDa filter (Pall, Vienna, Austria). The solution was centrifuged 2 × 20 min at 10,000 × *g.* The proteins were reduced with 200 mM dithiothreitol (37°C, 30 min) and alkylated with 500 mM iodoacetamide (37°C, 30 min) on the filter. After two washes with 100 µl of 50 mM TRIS, digestion was carried out using a trypsin/lysC mix in a ratio of 1:25 (protease: protein) overnight. Digested peptides were recovered with 3 × 50 µl of 50 mM TRIS and acidified with 1 µl concentrated trifluoroacetic acid (TFA). Before LC-MS analysis, peptide extracts were desalted and cleaned up using C18 spin columns (Pierce/Thermo Fisher Scientific, Waltham, Mass, USA) according to the manufacturer’s protocol. The dried peptides were redissolved in 300 µl of 0.1% TFA of which 3 µl was injected onto the LC-MS/MS system.

### 2.5 LC-MS/MS

Mass spectrometry analyses were performed on a LC-MS/MS system consisting of a nano-HPLC UltiMate 3000 RSLC (Thermo Fisher Scientific) directly coupled to a high-resolution Q Exactive HF Orbitrap^®^ mass spectrometer (Thermo Fisher Scientific) using a nano-ESI ion source.

Sample pre-concentration and desalting were accomplished with a 5-mm Acclaim PepMap μ−Precolumn (300-µm inner diameter, 5-µm particle size, and 100-Å pore size; Dionex/Thermo Fisher Scientific). For sample loading and desalting, 2% acetonitrile in ultra-pure H_2_O with 0.05% TFA was used as a mobile phase with a flow rate of 5 µl/min. Separation of peptides was performed on a 25-cm Acclaim PepMap C18 column (75-µm inner diameter, 2-µm particle size, and 100-Å pore size; Thermo Fisher Scientific) with a flow rate of 300 nl/min. The gradient started with 4% B (80% acetonitrile with 0.08% formic acid) for 7 min and increased to 31% in 60 min and to 44% in additional 5 min. It was followed by a washing step with 95% B. Mobile Phase A consisted of ultra-pure H_2_O with 0.1% formic acid.

The MS full scans were performed in the ultrahigh-field Orbitrap^®^ mass analyser in the range m/z 350–2,000 with a resolution of 60,000, the maximum injection time (MIT) was 50 ms, and the automatic gain control (AGC) was set to 3e^6^. The top 10 intense ions were subjected to Orbitrap^®^ for further fragmentation via high energy collision dissociation (HCD) activation over a mass range between m/z 200 and 2000 at a resolution of 15,000 with the intensity threshold at 4e^3^. Ions with charge states +1, +7, +8, and >+8 were excluded. Normalized collision energy (NCE) was set at 28. For each scan, the AGC was set at 5e^4^ and the MIT was 50 ms. Dynamic exclusion of precursor ion masses over a time window of 30 s was used to suppress repeated peak fragmentation.

### 2.6 Mass spectrometry data processing

The database search was performed using the Proteome Discoverer Software 2.4.305 (Thermo Fisher Scientific). The protein databases were downloaded from the UniProt homepage (http://www.uniprot.org) for the following species: *Cystoisospora suis* (taxonomy ID 483139) and *Sus scrofa* (taxonomy ID 9823). Additionally, to the combined UniProt databases, the common contaminant database cRAP was used (https://www.thegpm.org/crap/). Search settings were as follows: 10-ppm precursor mass tolerance and 0.02-Da fragment mass tolerance; dynamic modifications allowed were oxidation of methionine as well as the N-terminal protein modifications acetylation, methionine loss, and the combination of both, and static modification carbamidomethylating on cysteine. Only proteins with at least two identified peptides were reported. Intensity-based label-free quantification was applied to compare protein abundance in the experiments. Using Proteome Discoverer Software, abundance raw values were generated from mass spec raw files. Normalization to total peptide amount was performed within the software before abundance values were exported for further statistical analysis.

The mass spectrometry proteomics data have been deposited to the ProteomeXchange Consortium (http://proteomecentral.proteomexchange.org) via the PRIDE partner repository (Perez-Riverol et al., 2021) with the dataset identifier PXD045050.

### 2.7 Identification and analysis of differentially expressed proteins

All statistical analyses were performed in R v4.2.1 R Core Team (2022).

We filtered for 1,106 *C. suis* proteins that were quantifiable in a minimum of 75% of our samples (at least 30 out of our 40 measured samples derived from four biological replicates × two technical replicates × five timepoints, see **Figure S3**). To handle missing data, we employed a k-nearest neighbors (kNN) imputation algorithm. The Euclidean distances for this algorithm were computed as averages derived from non-missing quantifications among the 10 nearest neighbors. The imputation was executed using the multi.impute function from the mi4p package, version 1.0 (Chion et al., 2022). Both biological and technical replicates were imputed in a combined manner. For evaluating the imputation performance, the normalized root mean squared error (NRMSE) was calculated to be 0.232. The impute::Rmse(norm = TRUE) function was utilized for this calculation. To generate the NRMSE, we used a dataset containing 654 proteins that were quantifiable across all 40 samples. A subset, comprising 25% of this dataset, was masked using the imputeR:SimIm function (Feng et al., 2020). Subsequently, the masked data points were imputed using the impute::impute.knn(k=10) function for verification purposes (Hastie et al., 2023).

We performed principal component analysis on these 1,106 (centered and scaled) proteins after imputation using function *prcomp* in package *factoextra version 1.0.7* and visualized them with function *fviz_pca_ind* (Kassambara and Mundt, 2020). Differential protein expression analysis was performed with function *dream* in package *variancePartition* version 1.28.0 (Hoffman and Roussos, 2021), on log2-transformed, normalized LC-MS/MS quantifications after adding a constant of 1 via univariate linear mixed-effect model fitting timepoint of development as a fixed categorical effect with factor levels day 7, 8, 10, 12, and 14. The hierarchical covariance structure in our data, *aka* two technical replicates per biological replicate and timepoint and repeated measures of our four biological replicates across the five developmental stages, was modelled with a maximal random slopes approach to control the type one error rate (Barr et al., 2013).

We fitted a random intercept for biological replicate and dummy-coded, centered, random slopes for days 8 to 14. We further added a random intercept of date of experiment as precaution to account for possible batch effects. We used maximum likelihood estimation by setting option REML to “false”. We tested all 10 possible pairwise contrasts among the five timepoints with function *getContrast*. We requested moderated t-tests via function *eBayes* and verified that linear model assumptions were met for the residuals and random effects of a randomly chosen set of proteins.

Proteins with at least one contrast significant at a global 5% false discovery rate (FDR) and absolute log2 fold change > 1 were considered significantly differentially expressed. Significant proteins were subjected to the pattern detection algorithm used by function *degPatterns* in package *DEGreport version 1.34.0* (Pantano, 2023), sorted according to their cluster membership (largest to smallest cluster), visualized, row wise z-transformed, as unclustered heatmap, and produced with package *pheatmap version 1.0.12* (Kolde, 2012). For a more detailed information about validation of the method, see **supplemental material and method section**.

### 2.8 Gene annotation analyses

Protein annotations available on www.toxodb.org were used for *C. suis* proteins described in this study. The identification of potential homologues of *C. suis* hypothetical proteins was also carried out using BLAST analyses on www.toxodb.org and https://blast.ncbi.nlm.nih.gov/blast/Blast.cgi, employing an E-value cut-off of <0.0005. To explore the broader biological context of the identified genes, we used the Kyoto Encyclopaedia of Genes and Genomes (KEGG) database on https://www.genome.jp/kegg/kegg2.html.

## 3 Results

### 3.1 Primary results of LC-MS/MS, protein quantification, and hierarchical clustering analysis

To understand the proteomic changes reflecting the functional differences across the various life stages of *C. suis*, we applied LC-MS/MS analysis at five time points of *in vitro* development to quantify the protein levels and compare the expression at the different time points of parasite development. IPEC cells were infected with freshly excysted sporozoites, and culture supernatants containing developed parasite stages were harvested at different time points, day 7 after infection, designating the peak of asexual stages (early merozoites) in the absence of sexual stage, day 8 after infection with late merozoite considered to be sexually committed and early sexual stages, day 10 after infection with a mixture of late merozoites and immature and mature sexual stages, day 12 with mainly mature sexual stages and oocysts, and day 14 with mostly oocysts. Total protein was extracted from four biological replicates with two technical sub-replicates each for each time point. In total, 5,258 proteins were identified with at least two identified peptides of which at least one was unique per protein. After filtering out *S. scrofa*, the four biological replicates yielded a combined dataset of 1,324 proteins assigned to the *C. suis* genome.

### 3.2 Functional analysis of the protein dataset

**Figure 1:**
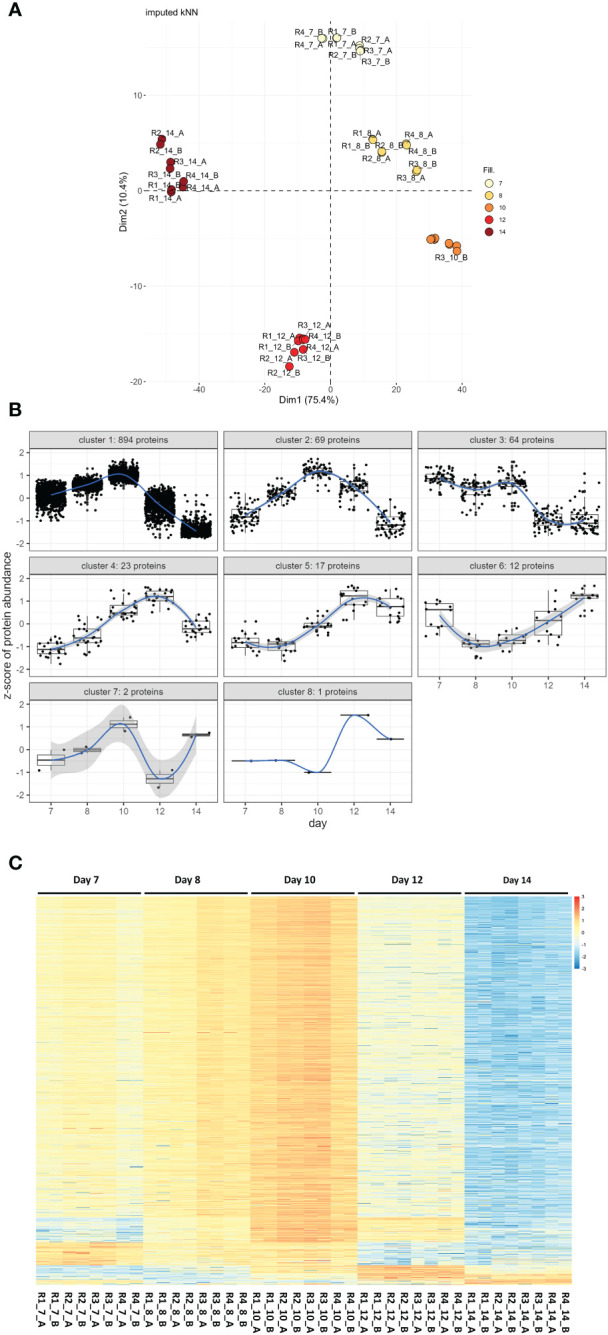
Proteome wide expression patterns across five *C*. *suis* developmental stages. **(A)** Principal component analysis based on log2 transformed, scaled, and centered, proteins after kNN imputation for missing data. Samples R1 to 4 with two technical sub-replicates _A and _B are colored according to their developmental stages day 7 to day 14. **(B)** Detected patterns of expression changes over developmental time detected for the 1,082 proteins that were statistically significant at global 5% FDR and a minimum log2 fold change *>=|1|* in at least one of the 10 contrasts tested per protein among the 1,106 proteins subjected to hypothesis testing after imputation (1,082/1,106 = 97.8% of proteins). **(C)** Unclustered heatmap showing row-wise z-transformed, protein expression across five developmental stages (day 7 to day 14) sorted according to their expression pattern. Each row represents a protein, higher than average expression in red, average expression white, less than average expression in blue. Each column represents a sample, R1–R4 are four biological replicates each with two technical sub-replicates (_A and _B).

Of the set of 1,324 proteins, 1,106 could be quantified in at least 75% of all samples. Missing data for these were imputed using kNN methodology. Principal component analysis on these 1,106 proteins showed perfect clustering according to their developmental stages along the first and second axes, explaining 75.4% and 10.4% of the variance, respectively (**Figure 1A**). These 1,106 proteins were then subjected to hypothesis testing. There were 1,082 proteins identified as significantly differentially expressed in at least one of the 10 tested contrasts at a global FDR cut-off of 5%, and with a minimum absolute log2 fold change of one (DEPs). These 1,082 proteins were subjected to a pattern detection algorithm with detected patterns shown in **Figure 1B** and then sorted according to their pattern-cluster membership (**Figure 1B**).

Their protein abundance is shown as a heatmap in **Figure 1C**. The number of differentially up- or downregulated proteins between two time points ranged from one to 1,010 (**Table 1**). The protein identification, description, and protein abundance levels for each of these proteins, at each developmental stage, are provided in **Table S1**.

**Table 1:** Summary table of the differential expression analysis of 1106 tested *C. suis* proteins, contrasting day 8-late merozoites (LMe), day 10-immature sexual stages (ISS) and day 12-mature sexual stages (MSS), and day 14-unsporulated oocysts (UO) compared to day 7-early merozoites (EMe), early vs late sexual stages and late sexual stages compared to oocysts showing the number of significantly up- or downregulated contrast at 5% FDR with a minimum effect size of absolute log2 fold change > 1 and the number of not differentially expressed proteins.

1,106 proteins > significant at 5% FDR and a minimum effect size of abs(log2(FC)) >1										
**Contrast**	**C_7.8**	**C_7.10**	**C_7.12**	**C_7.14**	**C_8.10**	**C_8.12**	**C_8.14**	**C_10.12**	**C_10.14**	**C_12.14**
**Contrast of stages**	**EMe vs LMe**	**EMe vs ISS**	**EMe vs MSS**	**EMe vs UO**	**LMe vs ISS**	**LMe vs MSS**	**LMe vs UO**	**ISS vs MSS**	**ISS vs UO**	**MSS vs UO**
**Down**	13	8	376	863	1	589	953	953	1010	764
**Not sig**	862	425	622	215	953	479	103	127	81	330
**Up**	231	673	108	28	152	38	26	21	15	12

**Figure 2:**
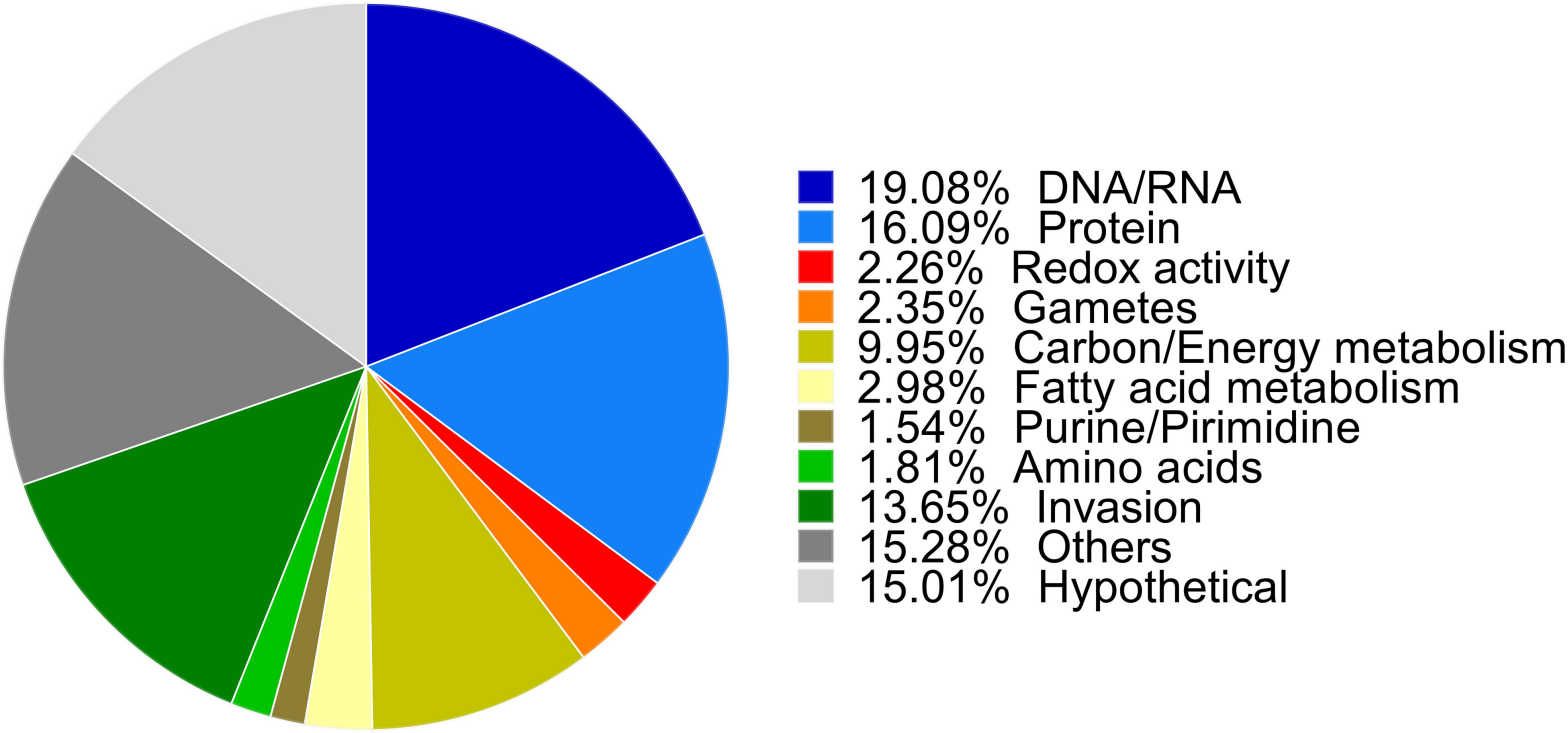
Biological functions of up- and downregulated proteins. Proteins were classified into functional categories. The biological functions were assigned manually based on GO biological process annotations provided by ToxoDB, annotations in the KEGG Pathway database for *T. gondii*, independent BLAST homology and previously published annotations and are shown as their relative proportions in the pie chart.

Proteins were classified into functional categories combining the Gene Ontology (GO) predictions for *C. suis* and *T. gondii* orthologues available in ToxoDB, according to annotations for *T. gondii* of the KEGG pathway database, BLAST homology searches or exploiting information contained in recent literature. In total, we were able to classify 70% of the 1,082 proteins into different categories. Functional annotation unveiled the most numerous functional categories included proteins involved in general macromolecule metabolism (30%), DNA and RNA processing (19%), and proteins related to the cell invasion process (13%). As expected, 15% were described as hypothetical proteins, and about 15% of the proteins were predicted to have diverse functions with undefined roles in parasite biology, e.g., membrane components or metal binding proteins (**Figure 2**).

### 3.3 Cellular metabolism

**Figure 3:**
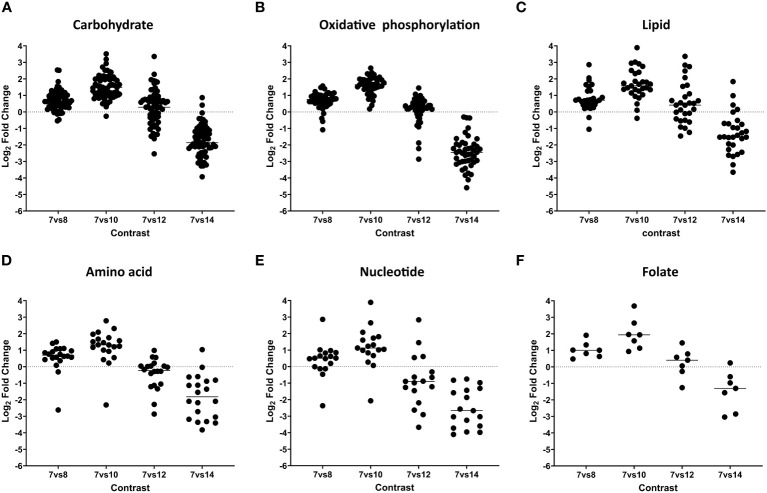
Dynamic expression pattern according to protein abundance. Panels **(A–F)**, show protein expression differences between developmental stages. Early merozoites (day 7) versus late merozoites (day 8), versus sexual stages (day 10), mature sexual stages (day 12), and oocysts (day 14) according to their functional classification. Every dot represents a protein that was identified as significantly differentially expressed at global 5% FDR and an absolute log2 fold change >1 in at least one contrast.

The proteomic analysis revealed 110 enzymatic components involved in carbon metabolism processes and 45 proteins participating in the mitochondrial electron respiratory chain (ETC) with higher expression levels during asexual and sexual stages, but not in unsporulated oocysts (**Figure 3**; **Tables 2**, **S2**). On day 12 of cultivation, sexual stages showed elevated expression of enzymes related to the tricarboxylic acid cycle (TCA). Specifically, branched-chain α-keto acid dehydrogenase E1 (BCKDH; CSUI_009934) in the mitochondrion and the pyruvate dehydrogenase complex (PDHC; CSUI_004282, CSUI_001749) in the apicoplast exhibited one- to threefold increased expression compared to merozoites. Additionally, increased levels of pyruvate carboxylase (CSUI_009794, CSUI_005195, CSUI_010742) and malate dehydrogenase (CSUI_010097) were observed, converting pyruvate to oxaloacetate and malate to oxaloacetate, respectively. Oxaloacetate, together with acetyl-CoA, serves as a substrate for the conversion to citrate by the action of citrate synthase (CSUI_001544, CSUI_006457), which was also increased on day 12. However, no significant increase in enzymes related to gluconeogenesis, phosphoenolpyruvate production, gamma-aminobutyric acid metabolism, or lactate metabolism was detected on day 12.

**Table 2:** List of proteins identified in this study. Table legend: They are listed along with their annotation number in ToxoDB, protein name, biological function, and protein abundance (LogFC), in each comparison. Early merozoites (day 7, C7) versus late merozoites (day 8, C8), immature sexual stages (day 10, C10), mature sexual stages (day 12, C12) and unsporulated oocysts (day 14, C14). Negative values indicate a downregulation.

ProbeID	Protein name	Function	Protein abundance (LogFC)			
			C 7_8	C 7_10	C 7_12	C 7_14
CSUI_004419	Glucosamine-fructose-6-phosphate aminotransferase	Amino sugar	1.201	3.190	1.461	-1.188
CSUI_003865	Phosphoglycerate kinase	Glycolysis	1.833	2.933	2.279	-0.732
CSUI_006394	Triosephosphate isomerase	Glycolysis/fructose pathway	1.190	2.157	1.864	0.868
CSUI_004282	Pyruvate dehydrogenase complex subunit pdh-e2	Pyruvate metabolism	2.519	3.515	3.358	-0.589
CSUI_009934	Branched-chain alpha-keto acid dehydrogenase e1 component beta	Pyruvate metabolism	0.814	2.043	1.950	-0.467
CSUI_009794	Pyruvate carboxylase	Pyruvate metabolism	1.056	2.404	1.901	0.413
CSUI_001749	Dihydrolipoamide dehydrogenase	Pyruvate metabolism	1.331	2.525	1.808	-1.112
CSUI_005195	Pyruvate carboxylase	Pyruvate metabolism	1.400	2.519	1.366	-3.224
CSUI_010742	Pyruvate carboxylase	pyruvate metabolism	1.083	2.048	0.896	-1.739
CSUI_006072	Pyruvate dehydrogenase complex subunit pdh-e3ii	Pyruvate metabolism	1.020	2.064	0.632	-2.088
CSUI_000883	Acetyl-carboxylase acc1	Pyruvate metabolism	0.564	1.259	0.557	-1.531
CSUI_006457	ATP citrate synthase	TCA	1.345	1.661	1.339	-0.413
CSUI_001544	Citrate synthase	TCA	1.287	2.215	1.262	-1.530
CSUI_010097	malate dehydrogenase	TCA	0.485	1.075	1.041	-0.876
CSUI_008592	Dihydroorotate dehydrogenase	Complex I/pyrimidine/folate metabolism	1.018	2.653	1.450	-0.970
CSUI_001762	Short chain dehydrogenase reductase family protein	Fatty acid biosynthetic process	0.261	0.844	2.481	0.982
CSUI_010330	Acyl carrier protein	Fatty acid biosynthetic process	1.514	2.756	2.079	-0.972
CSUI_002612	3-ketoacyl-(Acyl-carrier-protein) reductase	Fatty acid biosynthetic process	1.263	2.319	1.543	-1.414
CSUI_003216	Short chain dehydrogenase reductase family protein	Fatty acid biosynthetic process	-0.339	0.102	1.098	0.420
CSUI_000890	Beta-ketoacyl-[acyl-carrier-protein] synthase I	Fatty acid biosynthetic process	0.478	1.580	0.536	-2.696
CSUI_001389	Enoyl-acyl carrier reductase enr	Fatty acid biosynthetic process	0.712	1.859	0.410	-2.577
CSUI_003655	Strictosidine synthase subfamily	Glycerophospholipid met	2.862	3.891	2.835	-0.752
CSUI_007585	Phospholipase	Glycerophospholipid met	1.663	2.951	3.366	1.835
CSUI_002440	Acyltransferase domain-containing protein	Glycerophospholipid met	0.626	1.676	2.742	-0.013
CSUI_005022	Phosphatidylserine decarboxylase	Glycerophospholipid met	2.059	3.019	1.617	0.151
CSUI_006992	Acyltransferase domain-containing protein (ATSI)	Glycerophospholipid met	0.428	1.460	0.675	-0.695
CSUI_002060	Histone H4	Chromatin complex	2.027	2.915	2.968	1.199
CSUI_002076	Histone H3	Chromatin complex	1.996	2.956	2.893	1.131
CSUI_008436	Protein-serine/threonine kinase	Chromatin complex	2.176	4.096	2.851	0.847
CSUI_003118	Methyltransferase	Chromatin complex	0.905	2.578	2.575	0.192
CSUI_007420	RuvB-like helicase	Chromatin complex	1.245	2.313	1.634	1.236
CSUI_007597	histone H2AX	Chromatin complex	1.346	2.241	1.457	-0.956
CSUI_007088	RuvB family 2 RuvB family 2 protein	Chromatin complex	1.045	1.926	1.427	0.416
CSUI_002225	Histone H2Ba	Chromatin complex	0.883	1.707	1.376	-0.203
CSUI_005845	Protein-serine/threonine kinase	Chromatin complex	0.718	1.573	0.913	-3.152
CSUI_010473	Histone H2BZ	Chromatin complex	0.928	1.835	0.887	-1.022
CSUI_000278	Lysine–tRNA ligase	Translation-tRNA aminoacylation	1.062	1.164	2.007	1.081
CSUI_001201	Peptidyl-tRNA hydrolase	Translation-tRNA aminoacylation	2.706	2.856	1.408	1.013
CSUI_004846	Arginyl-tRNA synthetase	Translation-tRNA aminoacylation	0.190	0.679	1.083	1.294
CSUI_000414	Mandelonitrile lyase	Oxidation-reduction process	-0.746	1.185	3.052	1.463
CSUI_008814	Peroxiredoxin prx3	Oxidation-reduction process	1.086	2.354	1.594	-0.975
CSUI_000048	Morn repeat-containing protein	Microgametes	2.469	3.378	3.858	1.490
CSUI_011133	Membrane occupation and recognition nexus protein morn1	Microgametes	0.171	0.975	1.349	0.641
CSUI_011036	Morn repeat protein	Microgametes	1.450	2.626	1.339	0.156
CSUI_009771	Tubulin beta chain	Microtubule-based process	0.732	1.639	2.939	2.257
CSUI_010140	Microtubule-binding protein	Microtubule-based process	1.351	1.302	2.326	-0.030
CSUI_008696	Tubulin alpha chain	Microtubule-based process	0.989	1.818	1.508	-0.100
CSUI_006267	Tubulin beta chain	Microtubule-based process	0.662	1.727	1.362	-0.476
CSUI_006169	Tubulin beta chain	Microtubule-based process	1.245	2.267	1.243	-0.531
CSUI_003705	Alveolin domain containing intermediate filament imc12	Oocyst	4.308	7.338	6.839	3.160
CSUI_003616	Microneme protein	Oocyst	1.170	2.650	4.717	1.978
CSUI_000999	Alveolin domain containing intermediate filament imc7	Oocyst	2.403	4.825	4.496	1.464
CSUI_008977	Pan domain-containing protein	Oocyst	-1.735	2.527	4.234	2.527
CSUI_006641	Pan domain-containing protein	Oocyst	-1.396	2.613	4.200	2.498
CSUI_001473	TyRP_hypothetical protein	Oocyst	-1.525	3.393	4.031	0.869
CSUI_010687	Pan domain-containing protein	Oocyst	0.420	2.217	3.432	1.766
CSUI_006179	hypothetical protein-sag related	Oocyst	-0.153	1.499	3.342	1.390
CSUI_004248	Sag-related sequence srs26i	Oocyst	-0.119	3.367	3.288	0.877
CSUI_009196	Toxoplasma gondii family d protein	Oocyst	0.114	1.522	3.222	0.711
CSUI_007432	Pan domain-containing protein	Oocyst	0.000	1.555	2.719	0.545
CSUI_003908	Toxoplasma gondii family d protein	Oocyst	0.527	1.437	2.593	0.961
CSUI_010652	Alveolin domain containing intermediate filament imc7	Oocyst	-0.574	-0.187	1.365	1.495
CSUI_008449	Toxoplasma gondii family a protein	Oocyst	-0.721	0.218	1.287	0.622
CSUI_005717	Imc sub-compartment protein isp1	Oocyst	0.279	1.040	1.269	1.245

The analysis of lipid metabolism revealed the presence of 29 enzymes involved in fatty acid biosynthesis, elongation, and degradation during asexual and sexual stages (**Figure 3**; **Tables 2**, **S3**). On day 12, 11 enzymes were highly expressed, playing critical roles in the FASII pathway and glycerophospholipid metabolism. The FASII pathway involves a series of independent enzymes responsible for distinct steps in fatty acid (FA) chain elongation. The pyruvate dehydrogenase complex showed elevated expression on day 12, facilitating the conversion of pyruvate to acetyl-CoA, which is then converted to malonyl CoA by acetyl-CoA carboxylase (CSUI_000883) to fuel FA synthesis. The growing fatty acyl radicals bind to the acyl carrier protein (CSUI_010330), and fatty acyl elongation involves sequential reactions by enzymes β-ketoacyl-ACP synthase (CSUI_000890), β-ketoacyl-ACP reductase (CSUI_002612), and enoyl-ACP reductase (CSUI_001389). The FASII pathway results in the production of short FA chains, primarily C12:0, C14:0, and C16:0. Two acyltransferases (CSUI_002440 and CSUI_006992) were identified, indicating the capability of *C. suis* to synthesize and esterify activated FA chains onto a glycerol-3-phosphate backbone. This process leads to the formation of lysophosphatidic acid and, subsequently, phosphatidic acid (PA), representing the initial step in the *de novo* synthesis of all glycerophospholipids.

Regarding amino acid metabolism, our results show that there is a high expression of 21 key enzymes involved at day 10, but their levels decrease during later stages of development (days 12 and 14; **Figure 3**; **Table S4**). These proteins are involved in the synthesis of aspartate and asparagine (CSUI_008676, CSUI_006263, CSUI_003412, CSUI_001547), glutamine and glutamate (CSUI_010297, CSUI_10774, CSUI_10235, CSUI_003150), glycine and serine (CSUI_10078, CSUI_007993, CSUI_001515, CSUI_009280), proline (CSUI_001107, CSUI_000255), or tryptophan (CSUI_011414), as well as tyrosine transporters (CSUI_004056 and CSUI_006182).

Our analysis identified 17 enzymes associated with purine and pyrimidine pathways (**Figure 3**; **Tables 2**, **S4**). Additionally, it revealed the presence of seven enzymes involved in folate biosynthesis (**Tables 2**, **S4**). These enzymes displayed elevated expression levels during asexual and immature sexual stages and decreased levels in unsporulated oocysts except for CSUI_008592.

### 3.4 Processing of genetic information

**Figure 4:**
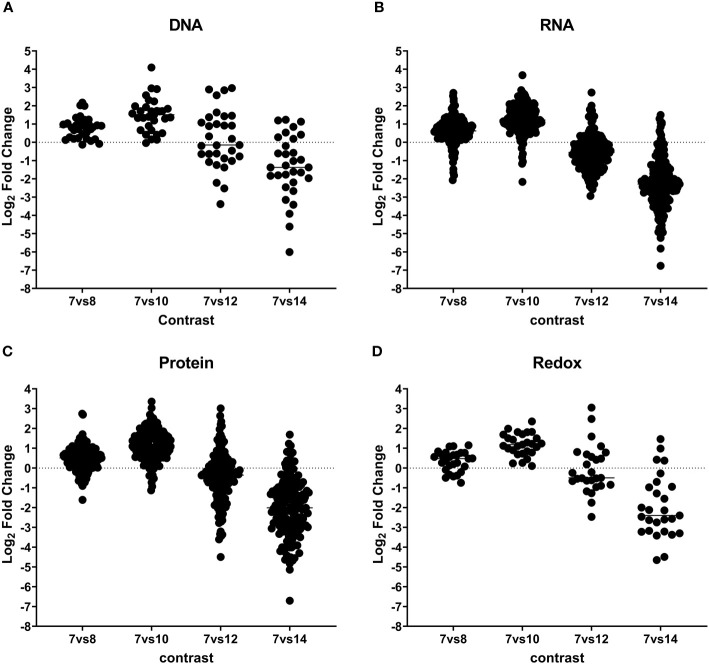
Dynamic expression pattern according to protein abundance. Panels **(A–D)** show protein expression differences between developmental stages. Early merozoites (day 7) versus late merozoites (day 8), versus sexual stages (day 10), mature sexual stages (day 12), and oocysts (day 14) according to their functional classification. Every dot represents a protein that was identified as significantly differentially expressed at global 5% FDR and an absolute log2 fold change >1 in at least one contrast.

The category of genetic information processing, encompassing core processes like DNA replication, transcription, and translation, is well represented in the *C. suis* proteome, comprising 213 proteins (see **Figure 4**; **Tables 2**, **S5**). These play diverse roles, including involvement in DNA replication, mitosis regulation, nucleosome formation, spliceosome complex, transcription factors, regulators of transcription and translation, ribosome formation, tRNA aminoacylation, and elongation factors crucial for protein translation. Notably, our analysis identified 15 proteins linked to the chromatin complex (H2AX; CSUI_007597, H2Ba; CSUI_002225, H2Bz; CSUI_010473, H3; CSUI_002076, H4; CSUI_002060, alongside several helicases and methyltransferases) with increased levels in sexual stages and oocysts (**Table S4**). By contrast, two histone proteins, H2AZ (CSUI_001093, CSUI_002075; **Table S5**), exhibited decreased levels during these stages. One methyltransferase (CSUI_003118) and two serine/threonine kinases (CSUI_008436, CSUI_005845) showed increased expression in sexual stages. Increased levels of proteins involved in RNA transcription, spliceosome assembly, ribosomal biogenesis, and RNA translation were found mostly on day 10 and then decreased again, except for three aminoacyl-tRNAs that were still upregulated on day 12 (CSUI_000278, CSUI_001201, CSUI_004846; **Table 2**).

In our analysis, we identified 53 proteins involved in protein binding, folding, and processing, 50 proteins related to protein transport, and 74 proteins associated with ubiquitination, proteolysis, and the proteasome complex (**Figure 4**; **Table S6**). We found increased levels of proteasome complex proteins on day 10 after which they decreased again.

### 3.5 Redox homeostasis

Our analysis revealed 29 proteins involved in oxidative homeostasis, all showing upregulation on day 10 during the transition from asexual to sexual stages (See **Figure 4**; **Tables 2**, **S7**). In the sexual stages, eight of these proteins remained upregulated, with mandelonitrile lyase (CSUI_000414) and peroxiredoxin 3 (CSUI_008814) exhibiting particularly high expression levels.

### 3.6 Proteins linked to sexual stages and cell invasion processes

**Figure 5:**
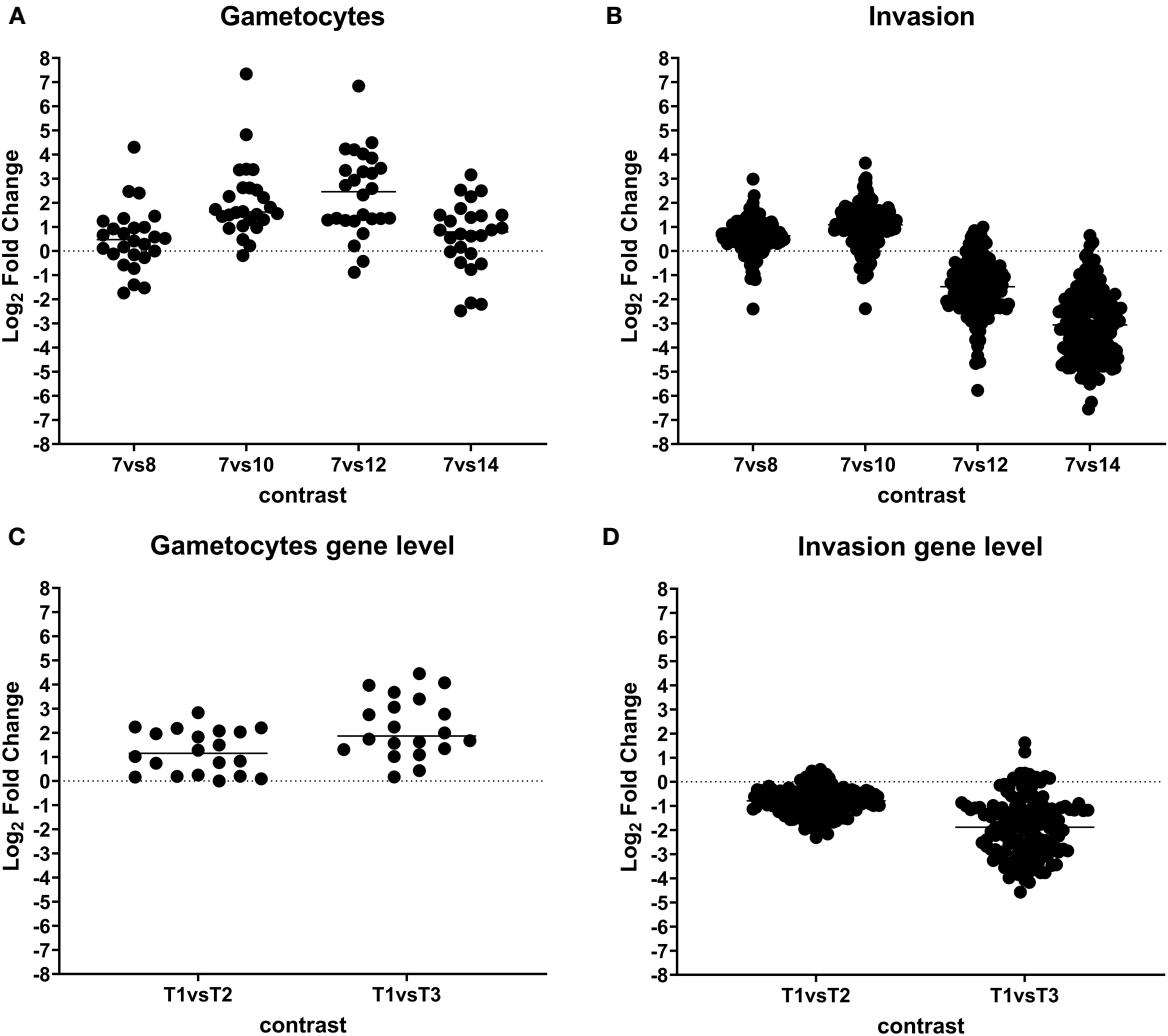
Dynamic expression pattern according to protein abundance. Panels **(A, B)** show protein expression differences between developmental stages. Early merozoites (day 7) versus late merozoites (day 8), versus sexual stages (day 10), mature sexual stages (day 12), and oocysts (day 14) according to their functional classification. Every dot represents a protein that was identified as significantly differentially expressed at global 5% FDR and an absolute log2 fold change >1 in at least one contrast. Panels **(C, D)** showing gene expression differences between developmental stages. T1: pool of days 6, 7, and 8 (early and late merozoites); T2: pool of days 9, 10, and 11 (late merozoites and immature sexual stages) and T3: pool of days 12, 13, and 14 (mature sexual stages and unsporulated oocysts).

Coccidia have two distinct sexual stages: macrogametes and microgametes. Most of the 26 proteins identified in relation to the development of macro- and microgametes (**Figure 5**; **Tables 2**, **S8**) are engaged in oocyst wall formation proteins (present in the developing macrogamete) and flagella/axoneme components (as part of the microgamete flagella) that were already identified in *C. suis*, *T. gondii*, and in *Eimeria* gametocytes and oocysts.

An enrichment of 122 proteins involved in invasion was found only on days 8 and 10 postinfection (**Figure 5**; **Table S9**), including 11 micronemal proteins and 3 PAN/Apple domains, 16 rhoptry and rhoptry neck proteins and 2 dense granules, 71 members of the large family of SAG-related (SRS) proteins, 15 members of the IMC, and 30 proteins involved in gliding motility.

We chose the proteins from both of these groups—invasion-related and gametocyte-associated proteins—to validate the accuracy of our methodology. To confirm our findings, we conducted a comparative analysis of this dataset with our prior RNA-seq data from different developmental stages of *C. suis*. The expression patterns of up- and downregulated genes in these two groups closely mirrored the results obtained with RNA-seq (**Figure 5**). As a deviation, the RNA data did not include six out of 26 proteins associated with sexual stages and 13 out of 150 proteins related to invasion.

## 4 Discussion

To gain insights into the functional variations during the transition from asexual to sexual stages of *C. suis*, it is crucial to identify, quantify, and compare the protein repertoire of each life cycle stage. To achieve this, we utilized LC-MS/MS analysis in this study to generate quantitative data on proteins expressed by *in vitro C. suis* stages. We identified a total of 1,082 individual proteins in the developmental stages of *C. suis*. Through BLAST, GO, and KEGG analyses, we observed that proteins involved in macromolecule metabolism, DNA and RNA processing, and proteins related to the cell invasion process were the predominant groups. We conducted a comparative analysis between the protein quantification patterns and our prior transcriptomic analysis of *C. suis* (Cruz-Bustos et al., 2022). Notably, the expression patterns of up- and downregulated genes within two functional groups (invasion and gametocyte-related proteins) were closely paralleled the RNA-seq results. The variability in expression levels in both methods could be attributed to the independent nature of the experiments and slight differences in their experimental designs. In the RNA-seq experiment, it was necessary to pool samples from 3 days to obtain sufficient material, whereas in the current experiments, we were able to analyse individual days. This dataset, thus, serves as a valuable resource for investigating stage-specific protein expression, providing insights into proteins displaying diverse expression levels across various developmental stages.

### 4.1 Cellular metabolism

Cellular metabolism is a complex web of chemical reactions occurring within cells, covering carbon, lipid, protein, purine, pyrimidine, and vitamin metabolism. Carbon metabolism is foundational to life and is profoundly reprogrammed in coccidian parasites during developmental stages, involving different sets of proteins to support adaptation and survival (Amiar et al., 2020; Xu et al., 2021). Over the past two decades, extensive research has been conducted on the energy metabolism of tachyzoites in *T. gondii* (Jacot et al., 2016). Glucose is taken up from the host cell by a glucose transporter located in the parasite’s plasma membrane. It is then catabolized into pyruvate, with some of it converted to lactate. Another portion of pyruvate enters the mitochondrion, producing acetyl-CoA, which, along with oxaloacetate, is converted into citrate to fuel the tricarboxylic acid (TCA) cycle (Jacot et al., 2016). The elevated expression of enzymes associated with the TCA cycle on immature sexual stages indicates the importance of TCA cycle activity for energy provision during sexual development. Interestingly, proteins participating in glycolysis and pentoses pathways were decreased in mature sexual stages (day 12 of cultivation) and drastically downregulated in unsporulated oocysts (day 14) compared to asexual and immature sexual stages (days 8 and 10). Tachyzoites of *T. gondii* can utilize glucose and glutamine as major carbon and energy sources to maintain their survival and virulence, with most of these nutrients used for ATP generation to facilitate gliding motility and host cell invasion (Garfoot et al., 2019). As with other Coccidia, *C. suis* asexual stages develop exclusively intracellularly, while sexual stages are found extracellularly in *in vitro* culture and probably also *in vivo* (Feix et al., 2021). As a result, the sexual stages can no longer rely on host-derived glucose or other hexoses as an energy source and become strictly dependent on alternative sources to meet their bioenergetic needs. We therefore assume that parasite metabolism must become independent of the host cell during this phase of development. Our findings strongly indicate that in *C. suis* mature sexual stages, the utilization of glucose through glycolysis is absent. Instead, the parasite relies on pyruvate metabolism as a bridge to the TCA cycle, providing the energy needed for further development. In contrast, sporulated oocysts of *T. gondii* rely on β-oxidation, importing lipids into the mitochondria or the peroxisomes, to produce acetyl-CoA for energy, rather than glycolysis (Tran et al., 2016; Moog et al., 2017). Six proteins involved in the degradation of lipids were downregulated in sexual stages including unsporulated oocysts, indicating that those stages do not primarily rely on this process to obtain acetyl-CoA for the TCA cycle. A speculative insight emerges, while pyruvate catabolism is essential for sexual development and fertilization support, its dominance diminishes after oocyst formation.

The lipid composition and changes during sexual development in coccidian parasites are poorly studied. The presence of a prokaryotic type II fatty acid synthesis (FASII) pathway in the apicoplast of *T. gondii* was discovered in recent works. This pathway is crucial during tachyzoite development but becomes dispensable after the conversion to the bradyzoite stage (Shunmugam et al., 2022). In *T. gondii in vitro* cultures, parasites exhibit increased lipid storage under starvation conditions (Amiar et al., 2020). Additionally, *T. gondii* strains adapted to extracellular environments sense low nutrient availability and compensate by up-regulating FASII-guided lipid synthesis (Vincent A. Primo et al., 2021). In the related apicomplexan *Plasmodium falciparum*, cholesteryl ester, diacylglycerol, and triacylglycerol are the major neutral lipid classes in mature gametocytes, serving as energy storage (Cruz-Bustos et al., 2022). Besides polysaccharide granules and wall-forming bodies, mature macrogametes of *T. gondii* contain numerous lipid droplets (Ferguson et al., 1975) that could serve as energy sources. In our previous transcriptomic analyses, 19 genes involved in lipid metabolism were upregulated in sexual stages (Cruz-Bustos et al., 2022), corroborating the proteomic data obtained here and suggesting a significant accumulation of storage lipids in sexual stages, likely involved in *C. suis* oocyst formation.

Apicomplexan parasites can synthesize various amino acids, while others, including seven out of nine essential amino acids—histidine, isoleucine, leucine, methionine, phenylalanine, tryptophan, and valine—as well as arginine and tyrosine, require uptake for the host cell (Krishnan and Soldati, 2021). The incomplete *de novo* pathway for synthesizing the remaining two essential amino acids lysine and threonine has been described in *T. gondii* and *Plasmodium* spp. (Krishnan and Soldati, 2021). In our study, we identified aspartate-semialdehyde dehydrogenase (CSUI_002776), involved in threonine synthesis, and dihydrodipicolinate synthase (CSUI_001859), involved in lysine synthesis. This strongly suggest the presence of a functional pathway for threonine and lysine biosynthesis also in *C. suis*.

Apicomplexa cannot synthesize purines and rely on salvaging them from their hosts. They can, however, synthesize pyrimidines *de novo* using amino acid precursors through a pathway shared with its host (Fox and Bzik, 2020). Dihydroorotate dehydrogenase (DHODH; CSUI_008592) expression was increased in the sexual stages. This enzyme plays a crucial role in producing uridine 5′-monophosphate (Fox and Bzik, 2020). It is responsible for converting dihydroorotate to orotate and generating electrons for coenzyme Q in the mitochondrion, contributing to the electron transport chain and ATP synthesis. Notably, other enzymes involved in pyrimidine biosynthesis exhibited reduced levels on day 12, while DHODH remained consistently expressed. This suggests that the increased DHODH level is primarily linked to its activity in the electron transport chain and ATP synthesis, rather than pyrimidine biosynthesis.

Sulphonamides are structural analogues of para-aminobenzoic acid (PABA) and competitively inhibit dihydropteorate synthetase (DPS), an enzyme involved folic acid synthesis (Bayly and Macreadie, 2002). Sulphonamides inhibit the development of asexual stages, but no the formation of sexual stages. *Cystoisospora suis* develops very rapidly and once merozoites escapes sulphonamide action, the parasite may no longer be susceptible to this drug class in the sexual stage, which may explain the poor efficacy of this drug class against *C. suis* in single treatments (Joachim and Mundt, 2011).

### 4.2 Posttranslational modifications

Posttranslational modifications are common in chromatin-associated proteins, particularly histones (Kim, 2018). Here, we also investigated changes in histone modifications in *C. suis* during different developmental stages. Focussing on identifying the histone units and histone modification enzymes with altered levels in sexual stages, we found an increased expression of chromatin-associated proteins in sexual stages and oocysts. This concerns 15 proteins linked to the chromatin complex (e.g., H2AX, H2Ba, H2Bz, H3, H4, along with helicases and methyltransferases), showing increased expression in sexual stages and oocysts (**Table S4**). Conversely, histone proteins H2AZ exhibited decreased levels during these stages. Chromatin-associated proteins, particularly histones, undergo posttranslational modifications (Kim, 2018). Histone modifications were further explored, referencing to *T. gondii* where nucleosomes consist of a conserved octamer with four histones, two H2A-H2B dimers and a (H3-H4)2 tetramer, exhibiting stage-specific characteristics (Vanagas et al., 2019). H2B is encoded by two separate genes. H2BA is mainly expressed in tachyzoites, while H2Bz (previously known as H2Bv) is restricted to sexual stages (Dalmasso et al., 2006). Additionally, there are two variants of H2A, H2A.X, which shares only 91% identity with canonical H2A, and H2A.Z, the most conserved histone variant across species. H2A.Z and H2B.Z are enriched at transcription start sites, while the H2A.X variant plays a role in gene silencing and DNA repair (Dalmasso et al., 2009; Bogado et al., 2014). Transcriptional and translational repression is an important regulatory mechanism in gene expression regulation (Zhang et al., 2013). A methyltransferase and two serine/threonine kinases demonstrated elevated expression in sexual stages. Previous studies have characterized histone modifications affecting gene expression, such as acetylation, methylation, and phosphorylation, in apicomplexan parasites (Vanagas et al., 2019). Histone methylation in *T. gondii* is associated with heterochromatin and the silencing of genes, while acetylation is known to be involved in gene transcription (Kim, 2018). Our findings suggest that these histone complexes and associated proteins may be involved in silencing genes expressed only in asexual stages while activating genes specific to sexual development. This is consistent with our earlier transcriptomic analyses of *C. suis* development (Cruz-Bustos et al., 2022).

RNA processing, particularly splicing, is well-known, and alternative splicing plays a crucial role in gene regulation (Yeoh et al., 2019), facilitating proper developmental transitions during the conversion from asexual to sexual stages and onwards to oocysts. Our study identified three enzymes upregulated in sexual compared to asexual stages, lysine-tRNA ligase, peptidyl-tRNA hydrolase, and arginyl-tRNA synthetase. Aminoacyl-tRNA synthetases are essential key enzymes in protein translation, catalyzing the addition of amino acids to the cognate tRNA. The resulting aminoacylated tRNA, a crucial substrate for protein translation, is transported by elongation factors to the ribosome, where protein synthesis occurs (Gill and Sharma, 2023). In eukaryotes, arginyl-tRNA synthetase is also involved in the ubiquitination of proteins destined for degradation (Varshavsky, 2011), while lysine-tRNA ligase participates in β-lysine modification, such as posttranslational modifications of elongation factor P, in bacteria (Bullwinkle et al., 2013). Further investigation is required to understand the roles of these enzymes in protein synthesis and degradation in sexual stages of Coccidia.

### 4.3 Sexual related proteins

The life cycle of coccidian parasites involves stages with specialized functions. Apart from the function as fusion partner for microgametes, macrogametes are also critical in the synthesis and storage of materials necessary for later oocyst wall formation, including polysaccharides and lipids (Ferguson et al., 1975; Belli et al., 2006), while motile microgametes originate from microgamonts, characterized by nuclear division and the unique formation of flagella (Scholtyseck et al., 1972). Molecular characterization of macrogametes and oocysts in various coccidian parasites, such as *C. suis*, *T. gondii*, and *Eimeria* spp., has revealed specific gene expression patterns. Macrogametes express genes related to oocyst wall proteins, including proteases, oxidoreductases, peroxidases, glycosylation enzymes, and fatty acid metabolism enzymes. In contrast, microgametes exhibit increased expression of genes associated with axonemes, flagellar proteins, DNA condensation, and gamete fusion (Walker et al., 2015; Su et al., 2018; Ramakrishnan et al., 2019; Cruz-Bustos et al., 2022). In sexual stages, our study identified 13 upregulated proteases involved in oocyst wall formation, including aspartyl protease, serine protease, aminopeptidase, and seven subtilisins. In *Eimeria tenella*, subtilisins have been implicated in processing tyrosine-rich proteins during gametocyte development (Walker et al., 2015). Interestingly, proteasome activity was reduced during fertilization and oocyst formation, consistent with observations in *P. falciparum* gametocytes and zygote development (Ngwa et al., 2013). Oocyst wall proteins and gametocyte-specific proteins were previously characterized in *Eimeria* spp., *T. gondii*, and *C. suis* as the main protein constituents of the oocyst wall, and approximately 90% of it consist of cross-linked tyrosine-rich and cysteine-rich proteins (Ferguson et al., 2003; Belli et al., 2006; Possenti et al., 2010; Possenti et al., 2013; Salman et al., 2017; Cruz-Bustos et al., 2022). Here, we observed a corresponding increase in the expression of four cysteine-rich proteins (homologous to *T. gondii* proteins; Salman et al., 2017), as well as a tyrosine-rich protein (CSUI_001473) on day 12, consistent with our previous transcriptomic studies (Feix et al., 2020; Feix et al., 2021; Cruz-Bustos et al., 2022), and an increase of tyrosine transporter proteins on day 10. This finding leads us to speculate that the uptake of tyrosine, facilitated by two tyrosine transporters, and the subsequent formation of tyrosine- (and cysteine-) rich proteins play a crucial role in oocyst wall development also in *C. suis*. Microgametes, on the other hand, exhibited increased expression of proteins involved in MORN repeated domain structure and tubulin, essential for flagellar motility (Ferguson et al., 2008; Francia et al., 2016).

### 4.4 Invasion related proteins

Coccidian asexual stages display a strictly intracellular development, employing diverse strategies to facilitate cell adhesion, invasion, and intracellular survival. These invasive stages are characterized by specialized cellular structures and organelles closely attached to their membranes (Kato, 2018). Adaptive changes in cellular architecture are pivotal for invasion, replication, and egress in parasites. The inner membrane complex (IMC), a structural element composed of flattened alveolar sacs underlying the plasma membrane, plays a significant role in these morphological changes (Ferreira et al., 2021). This system is linked to a supporting cytoskeletal network and is vital for intracellular replication, motility, and host cell invasion (Harding and Frischknecht, 2020). In particular, the actin–myosin motor complex, known as the “glideosome,” is essential for cell motility and host cell seeking includes proteins like myosins, tubulins, actins, and glideosome-associated proteins (Boucher and Bosch, 2015). Surface antigens, mainly glycosylphosphatidylinositol (GPI)-anchored, play pivotal roles in *T. gondii* adhesion to host cells, immune evasion, and host specificity. This group includes SAG1-like and SAG2-like proteins, along with SAG-related surface antigens (SRS) (Lekutis et al., 2001). After attachment to the host cell, *Toxoplasma gondii* employs an array of proteins for cell invasion and regulation of host protein expression, including micronemal proteins (MICs), PAN/Apple domains, rhoptry and rhoptry neck proteins (ROPs and RONs), and dense granules (GRAs) (Dubremetz, 2007; Frénal et al., 2017; Dubois and Soldati-Favre, 2019; Attias et al., 2020). In merozoites and sexual stages (both residing in the intestinal cells of the feline final host of *T. gondii*), the expression of proteins such as rhoptries and dense granules is downregulated compared to the quickly replicating tachyzoites (Hehl et al., 2015; Ramakrishnan et al., 2019).

Here, all proteins involved in invasion of *T. gondii* were also identified in *C. suis* (**Table S9**), and their downregulation in sexual stages indicates reduced interactions with host cells and the host’s immune system. This is likely due to the unique characteristics of sexual stages, which swiftly progress from gamonts to oocysts and do not extensively reinvade cells. In line with our previous work on the development of *C. suis* (Cruz-Bustos et al., 2022), we showed that the sexual stages lack the machinery necessary for the invasion process, supporting the concept that these stages are extracellular and suggesting that the fertilization process (and hence oocyst formation) occurs outside the host cell, making these stages accessible targets for specific antibodies.

## 5 Conclusion

In this comparative proteomic analysis, we investigated the proteins involved in the transition from asexual to sexual stages in coccidian parasites. During sexual differentiation and oocyst formation, we observed significant changes in the expression of proteins related to cellular processes, metabolism, and host–pathogen interaction. These changes reflect the adaptation of sexual stages to a nutrient-poor and potentially stressful extracellular environment, with a focus on enzymes involved in metabolism and energy production.

The alterations in carbon metabolism may be linked to the transition from anaerobic conditions within the host cell to the aerobic development of late sexual stages and the oocyst outside the host cell. Additionally, our findings highlight the significance of histone modifications as essential epigenetic mechanisms in regulating gene expression during the complex life cycle of the Coccidia. Understanding the interplay between transcription factors and epigenetic modifications in these parasites could lead to the identification of potential targets for therapeutic interventions against *C. suis* infections (and probably against other Coccidia as well).

Our discoveries not only contribute to the comprehension of *C. suis* developmental biology but also offer valuable insights that could extend to other coccidian parasites. This work establishes *C. suis* as a model for comparative studies on coccidian parasites, especially the cyst-forming members of the family Sarcocystidae, opening new avenues for future research. Overall, our study greatly advances our understanding of coccidian parasites in general and may pave the way for the development of effective treatments and control strategies against these parasitic infections.

## Data availability statement

The datasets presented in this study can be found in online repositories. The names of the repository/repositories and accession number(s) can be found below: ProteomeXchange with identifier PXD045050.

## Ethics statement

All procedures in this study involving experimental animals were approved by the institutional ethics and animal welfare committee and the national authority according to §§26ff. of the Animal Experiments Act, Tierversuchsgesetz 2021—TVG 2012 under number 2021–0.030.760. All efforts were made to minimize the number of animals used for *C. suis* oocyst generation. All methods were performed in accordance with the guidelines and regulations approved by University of Veterinary Medicine Vienna and the national authority (Austrian Federal Ministry of Science, Health, and Economy). The study is reported in accordance with ARRIVE guidelines.

## Author contributions

TC-B: Conceptualization, Formal Analysis, Investigation, Methodology, Visualization, Writing – original draft, Writing – review & editing. MD: Data curation, Methodology, Writing – review & editing. AF: Investigation, Writing – review & editing. BR: Investigation, Writing – review & editing. KH: Conceptualization, Methodology, Writing – review & editing. ER-F: Conceptualization, Methodology, Writing – review & editing. AJ: Conceptualization, Funding acquisition, Project administration, Resources, Visualization, Writing – review & editing.

## Supplementary material

Supplementary Figure 1 Description of stages at each time point. **(A)** Schematic representation of the proposed *C. suis in vitro* developmental model. Oocysts are excreted in the faeces and sporulate in the environment. Under laboratory conditions, sporozoites released from oocysts by excystation invade the monolayer IPEC-1 cells and develop into merozoites. Merogony is followed by gamogony, in which the macro- and microgametes fuse to form a zygote and subsequently an oocyst. **(B)** Total amount of *C. suis* stages in *in vitro* cell culture. Values represent the mean from four biological replicates.

Supplementary Figure 2 Experimental design workflow for quantitative proteome analysis from *in vitro* asexual to sexual stages in *C. suis* using LC/MS-MS.

Supplementary Figure 3 Binary Heatmap. Binary heatmap produced with DEP::plot_missval (Zhang et al., 2018) of 1106 C*. suis* proteins after filtering for proteins that were measured in at least 75% of our samples. Black - protein quantifiable, white - quantification missing, rows are proteins, columns samples R1-R4 biological samples **(A, B)** technical sub-replicates. 7,8,10,12 and 14 timepoints.

Supplementary Figure 4 Dynamic expression patterns. Detected patterns of expression changes in 634 non-imputed proteins significant at a global 5% FDR, in at least one of the 10 contrasts tested per protein, with a log2 Fold Change >=|1| from a total of 654 proteins tested, that could be measured in all samples.

Supplementary Table 1 Differential expression of proteins in *C. suis*. Protein abundance and annotation of *C. suis* up- and downregulated proteins compared by five time points of parasite cultivation. The log2 fold change, average expression,”t” t-test statistic, P.Value” nominal uncorrected P-Value, “B” log-odds that that protein is differentially expressed, “z.std “ z score, “contrast” day 2^nd^ level. day 1^st^ level, “adj.P.Val “ false discover rate (FDR) multiple testing corrected P value, “s” significant and “n.s.” non-significant (“sign” ns if adj.P.Val >5%, “s” if adj.P.Val <5%), “sign_updown” significant at 5% FDR & effect size conditions if abs(logFC>1), day7-14 average expression on each day and comparison are listed.
