# Supplementary figures and images for: Unravelling the sexual developmental biology of Cystoisospora suis, a model for comparative coccidian parasite studies
Source: Front Cell Infect Microbiol. 2023 Oct 25;13:1271731. doi: 10.3389/fcimb.2023.1271731 (PMC10635411; doi:10.3389/fcimb.2023.1271731)

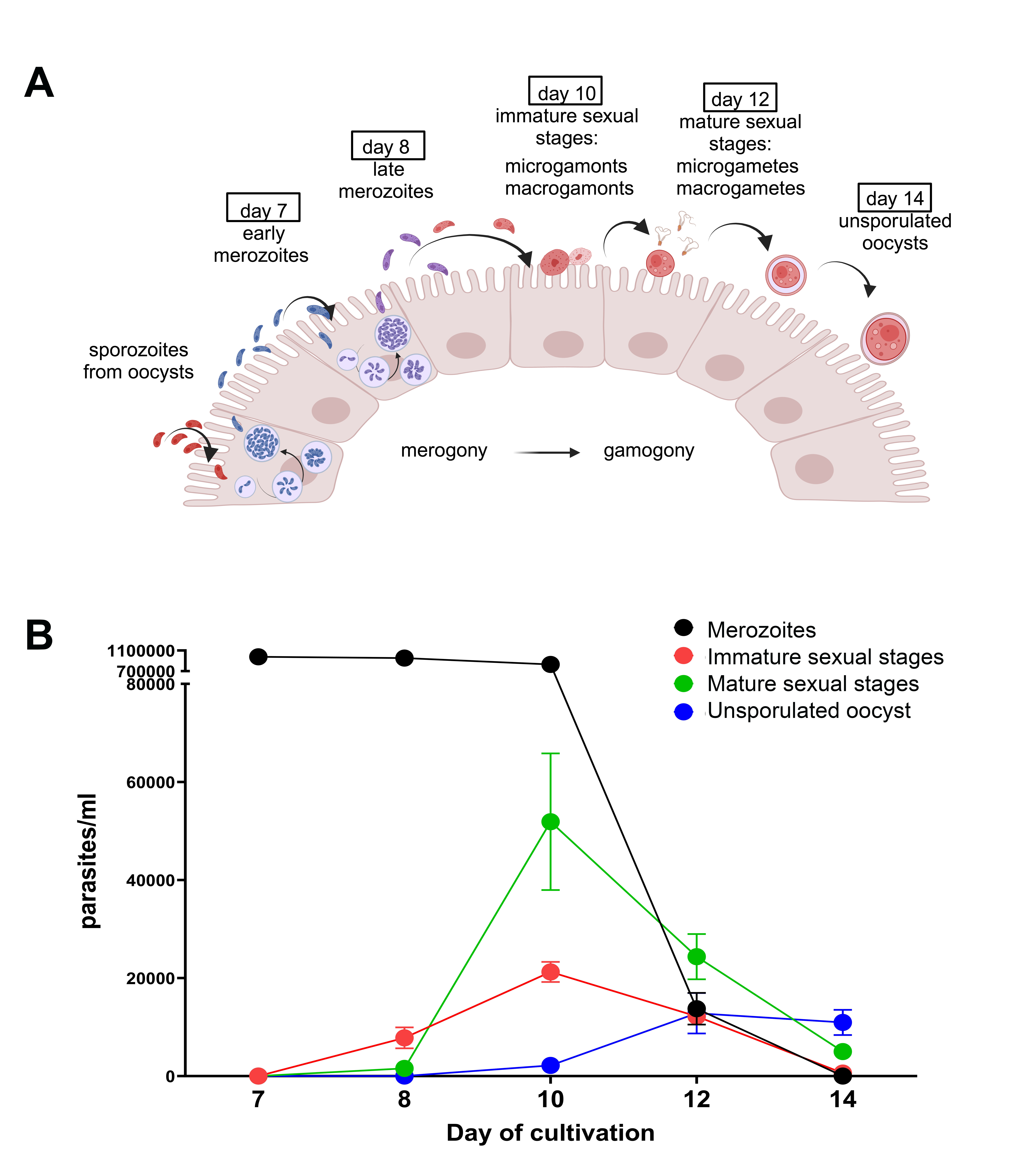

Supplement: Supplementary Figure 1 — Description of stages at each time point. (A) Schematic representation of the proposed C. suis in vitro developmental model. Oocysts are excreted in the faeces and sporulate in the environment. Under laboratory conditions, sporozoites released from oocysts by excystation invade the monolayer IPEC-1 cells and develop into merozoites. Merogony is followed by gamogony, in which the macro- and microgametes fuse to form a zygote and subsequently an oocyst. (B) Total amount of C. suis stages in in vitro cell culture. Values represent the mean from four biological replicates. [file Image_1.jpeg]

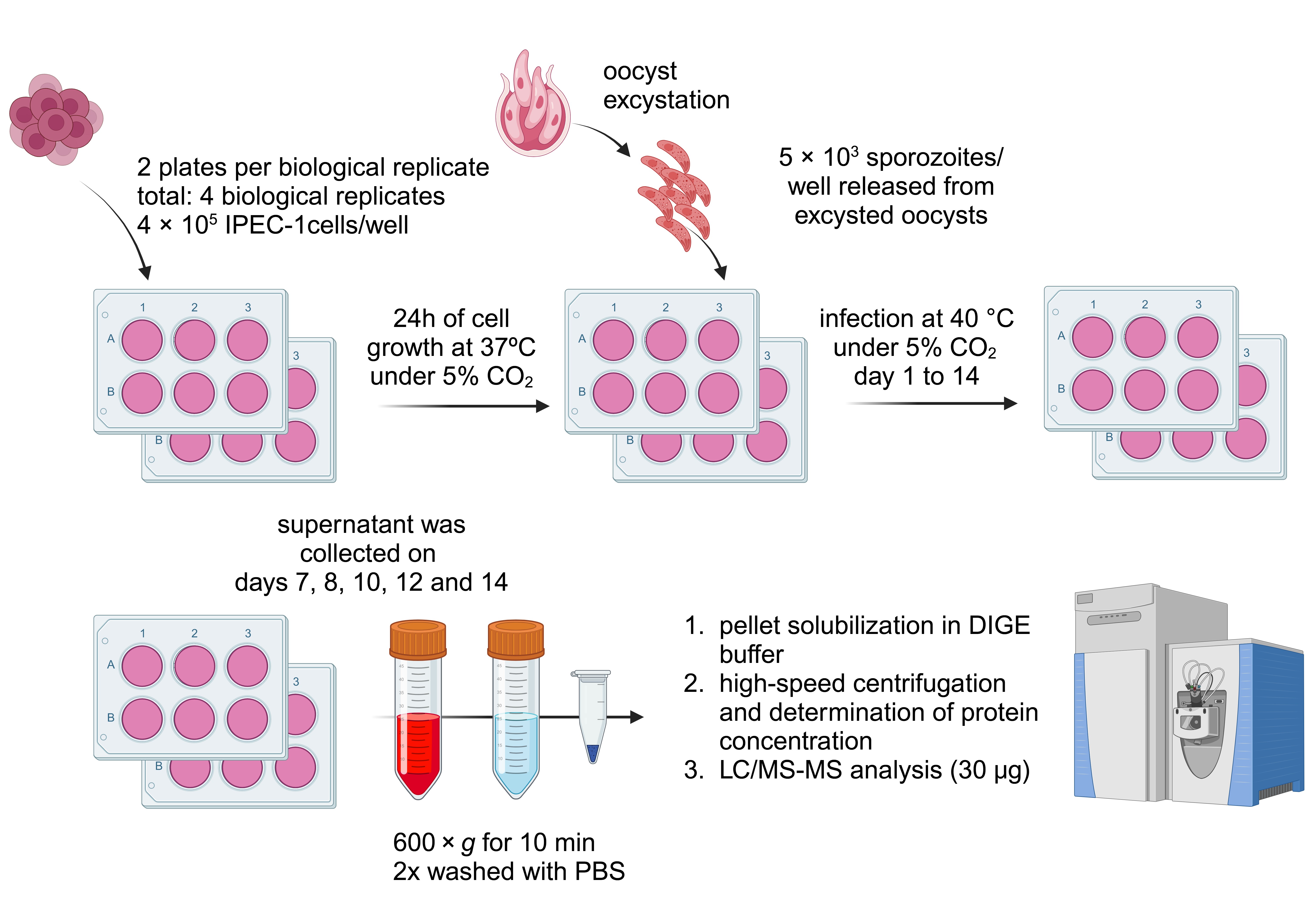

Supplement: Supplementary Figure 2 — Experimental design workflow for quantitative proteome analysis from in vitro asexual to sexual stages in C. suis using LC/MS-MS. [file Image_2.jpeg]

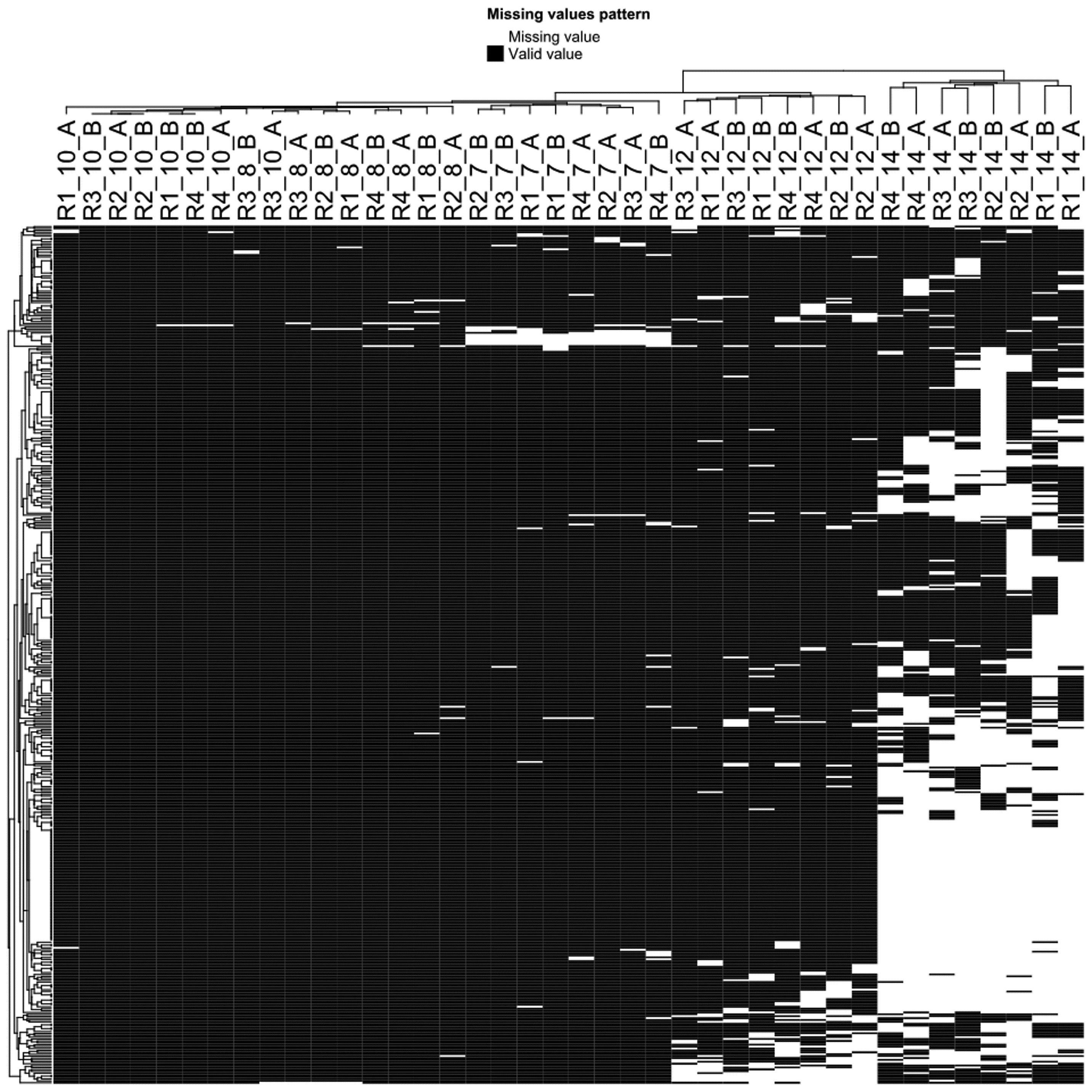

Supplement: Supplementary Figure 3 — Binary Heatmap. Binary heatmap produced with DEP::plot_missval (Zhang et al., 2018) of 1106 C. suis proteins after filtering for proteins that were measured in at least 75% of our samples. Black - protein quantifiable, white - quantification missing, rows are proteins, columns samples R1-R4 biological samples (A, B) technical sub-replicates. 7,8,10,12 and 14 timepoints. [file Image_3.jpeg]

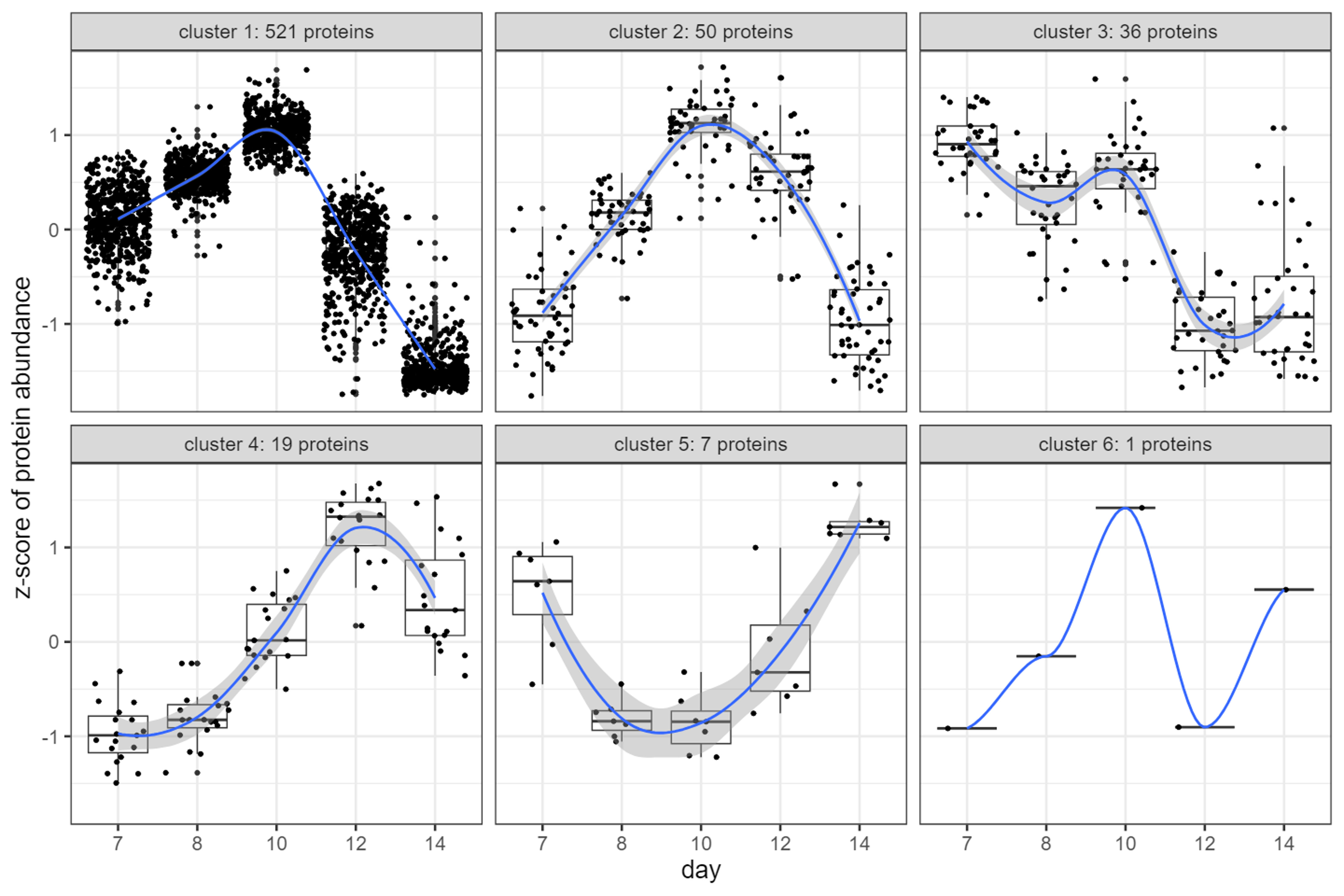

Supplement: Supplementary Figure 4 — Dynamic expression patterns. Detected patterns of expression changes in 634 non-imputed proteins significant at a global 5% FDR, in at least one of the 10 contrasts tested per protein, with a log2 Fold Change >=|1| from a total of 654 proteins tested, that could be measured in all samples. [file Image_4.jpeg]
