# Supplementary material for: Unravelling the sexual developmental biology of Cystoisospora suis, a model for comparative coccidian parasite studies
Source: Front Cell Infect Microbiol. 2023 Oct 25;13:1271731. doi: 10.3389/fcimb.2023.1271731 (PMC10635411; doi:10.3389/fcimb.2023.1271731)
Supplement: Supplementary file 6 [file Table_2.docx]

**Table S2. Proteins with putative roles in the central carbon and energy metabolism.** They are listed along with their annotation number in ToxoDB, protein name, biological function, and abundance (LogFC), in each comparison. Early merozoites (day 7, C7) versus late merozoites (day 8, C8), versus sexual stages (day 10, C10), mature sexual stages (day 12, C12) and oocysts (day 14, C14).

| **ProbeID** | **Protein name** | **Function** | **C 7_8** | **C 7_10** | **C 7_12** | **C 7_14** |
| --- | --- | --- | --- | --- | --- | --- |
| CSUI_004419 | glucosamine-fructose-6-phosphate aminotransferase | amino sugar | 1.201 | 3.190 | 1.461 | -1.188 |
| CSUI_000182 | Hexose transporter | glucose transport | 0.632 | 1.653 | 0.273 | -1.689 |
| CSUI_005659 | Glycosyl family 31 protein | glucose transport | 0.522 | 1.026 | -0.342 | -1.327 |
| CSUI_002771 | Fructose-bisphosphatase | fructose pathway | 0.583 | 1.136 | -1.043 | -2.158 |
| CSUI_000032 | Fructose-bisphosphatase | fructose pathway | 0.436 | 0.967 | -1.385 | -3.270 |
| CSUI_004045 | glycogen phosphorylase family | glycogen catabolic process | 0.040 | 0.775 | -0.435 | -2.009 |
| CSUI_000555 | Amylo-alpha | glycogen catabolic process | -0.036 | 0.495 | -1.475 | -2.112 |
| CSUI_003865 | Phosphoglycerate kinase | glycolysis | 1.833 | 2.933 | 2.279 | -0.732 |
| CSUI_003881 | Phosphoglycerate mutase | glycolysis | 0.568 | 1.494 | 0.221 | -1.969 |
| CSUI_003666 | Glyceraldehyde-3-phosphate dehydrogenase | glycolysis | 0.629 | 1.362 | 0.164 | -1.526 |
| CSUI_007933 | enolase | glycolysis | 0.479 | 1.482 | -0.177 | -2.104 |
| CSUI_001020 | Phosphoglycerate kinase | glycolysis | 0.449 | 1.269 | -0.281 | -2.207 |
| CSUI_007934 | enolase | glycolysis | 0.256 | 1.179 | -0.334 | -2.893 |
| CSUI_008370 | Glucose-6-phosphate isomerase | glycolysis | 0.289 | 1.156 | -0.335 | -1.983 |
| CSUI_000253 | Fructose-bisphosphate aldolase | glycolysis | 0.555 | 1.422 | -0.563 | -2.697 |
| CSUI_002763 | Pyruvate kinase | glycolysis | -0.087 | 0.617 | -0.888 | -0.886 |
| CSUI_008635 | Sedoheptulose--bisphosphatase | glycolysis | -0.068 | 0.314 | -1.303 | -1.335 |
| CSUI_006394 | Triosephosphate isomerase | glycolysis/fructose pathway | 1.190 | 2.157 | 1.864 | 0.868 |
| CSUI_008029 | Triosephosphate isomerase | glycolysis/fructose pathway | 0.663 | 1.577 | 0.002 | -1.514 |
| CSUI_000558 | phosphofructokinase pfkii | glycolysis/fructose pathway | 0.324 | 1.068 | -0.815 | -2.548 |
| CSUI_010801 | hexokinase | glycolysis/fructose pathway | 0.132 | 0.853 | -1.049 | -3.268 |
| CSUI_008434 | Glucose-6-phosphate 1-dehydrogenase (Fragment) | glycolysis/pentosa phosphate pathway | 0.629 | 0.890 | 0.319 | -0.793 |
| CSUI_009063 | Glucose-6-phosphate 1-dehydrogenase | glycolysis/pentosa phosphate pathway | 0.154 | 1.076 | -0.411 | -3.066 |
| CSUI_000469 | Phosphoglucomutase parafusin related protein | glycolysis/pentosa phosphate pathway | -0.129 | 0.669 | -0.893 | -2.667 |
| CSUI_002185 | Gpi16 gpi transamidase component protein | GPI-anchor biosynthesis | 1.504 | 2.156 | 0.717 | -0.958 |
| CSUI_000698 | hypothetical protein | N-glycan synthesis | 0.323 | 0.822 | -0.693 | -1.001 |
| CSUI_009070 | Transaldolase | pentose phosphate pathway | 1.172 | 2.179 | 0.803 | -2.961 |
| CSUI_006534 | 6-phosphogluconate dehydrogenase, decarboxylating | pentose phosphate pathway | 0.709 | 1.451 | 0.708 | -0.404 |
| CSUI_009225 | Transaldolase | pentose phosphate pathway | 0.683 | 1.543 | 0.584 | -1.660 |
| CSUI_009980 | Phosphoribosylpyrophosphate synthetase | pentose phosphate pathway | 0.625 | 1.608 | 0.549 | -1.867 |
| CSUI_001656 | Ribose-phosphate pyrophosphokinase | pentose phosphate pathway | 0.387 | 0.958 | -0.082 | -1.298 |
| CSUI_001025 | Transketolase | pentose phosphate pathway | 0.402 | 1.187 | -0.823 | -3.926 |
| CSUI_003623 | Ribose-5-phosphate isomerase | pentose phosphate pathway | -0.021 | 0.836 | -0.906 | -1.245 |
| CSUI_004282 | pyruvate dehydrogenase complex subunit pdh-e2 | pyruvate metabolism | 2.519 | 3.515 | 3.358 | -0.589 |
| CSUI_009934 | Branched-chain alpha-keto acid dehydrogenase e1 component beta | pyruvate metabolism | 0.814 | 2.043 | 1.950 | -0.467 |
| CSUI_009794 | Pyruvate carboxylase | pyruvate metabolism | 1.056 | 2.404 | 1.901 | 0.413 |
| CSUI_001749 | Dihydrolipoamide dehydrogenase | pyruvate metabolism | 1.331 | 2.525 | 1.808 | -1.112 |
| CSUI_005195 | Pyruvate carboxylase | pyruvate metabolism | 1.400 | 2.519 | 1.366 | -3.224 |
| CSUI_010742 | Pyruvate carboxylase | pyruvate metabolism | 1.083 | 2.048 | 0.896 | -1.739 |
| CSUI_006072 | pyruvate dehydrogenase complex subunit pdh-e3ii | pyruvate metabolism | 1.020 | 2.064 | 0.632 | -2.088 |
| CSUI_000883 | acetyl- carboxylase acc1 | pyruvate metabolism | 0.564 | 1.259 | 0.557 | -1.531 |
| CSUI_003451 | lactate dehydrogenase ldh1 | pyruvate metabolism | 0.626 | 1.441 | 0.288 | -1.316 |
| CSUI_005036 | Pyruvate dehydrogenase E1 component subunit alpha | pyruvate metabolism | 0.184 | 1.032 | 0.272 | -0.060 |
| CSUI_009417 | Pyruvate dehydrogenase E1 component subunit beta | pyruvate metabolism | 0.400 | 1.169 | 0.198 | -1.983 |
| CSUI_001676 | Pyruvate kinase | pyruvate metabolism | 0.680 | 1.131 | -0.175 | -1.097 |
| CSUI_003662 | Phosphoenolpyruvate carboxykinase (PEPCK) | pyruvate metabolism | 0.394 | 0.938 | -2.538 | -3.209 |
| CSUI_000956 | ribophorin i protein | protein glycosylation | 0.090 | 0.505 | -1.363 | -3.095 |
| CSUI_007407 | Glycan | sucrose pathway | -0.539 | -0.261 | -1.625 | -2.471 |
| CSUI_006457 | ATP citrate synthase | TCA | 1.345 | 1.661 | 1.339 | -0.413 |
| CSUI_001544 | Citrate synthase | TCA | 1.287 | 2.215 | 1.262 | -1.530 |
| CSUI_010097 | malate dehydrogenase | TCA | 0.485 | 1.075 | 1.041 | -0.876 |
| CSUI_010055 | Oxoglutarate dehydrogenase (Succinyl-transferring) e1 component | TCA | 1.247 | 2.124 | 0.951 | -1.075 |
| CSUI_003030 | Flavoprotein subunit of succinate dehydrogenase | TCA | 1.006 | 1.995 | 0.941 | -1.614 |
| CSUI_001565 | Fumarate hydratase | TCA | 1.013 | 1.883 | 0.765 | -1.980 |
| CSUI_001039 | Fad malate-dehydrogenase (Mdh-fad) | TCA | 1.060 | 1.960 | 0.759 | -1.632 |
| CSUI_002100 | Isocitrate dehydrogenase (NADP(+)) | TCA | 0.795 | 1.950 | 0.732 | -2.178 |
| CSUI_011378 | Dihydrolipoyllysine-residue succinyltransferase component of oxoglutarate dehydrogenase (Fragment) | TCA | 1.156 | 2.044 | 0.683 | -1.883 |
| CSUI_005867 | Oxoglutarate dehydrogenase (Succinyl-transferring) e1 component | TCA | 1.093 | 1.601 | 0.640 | -0.979 |
| CSUI_002732 | succinate dehydrogenase | TCA | 1.552 | 2.433 | 0.614 | -2.073 |
| CSUI_002305 | Succinate--CoA ligase [ADP-forming] subunit alpha, mitochondrial | TCA | 0.858 | 2.115 | 0.603 | -2.734 |
| CSUI_003925 | Succinate--CoA ligase [ADP-forming] subunit beta, mitochondrial | TCA | 0.962 | 1.904 | 0.602 | -2.439 |
| CSUI_006564 | Dihydrolipoyllysine-residue succinyltransferase component of oxoglutarate dehydrogenase | TCA | 0.959 | 1.991 | 0.416 | -2.414 |
| CSUI_002939 | aconitate hydratase acn irp | TCA | 0.716 | 1.672 | 0.282 | -1.943 |
| CSUI_009970 | Flavoprotein subunit of succinate dehydrogenase (Fragment) | TCA | 0.178 | 1.073 | -0.027 | -2.089 |
| CSUI_010237 | Citrate synthase i | TCA | -0.466 | 0.805 | -0.622 | -2.629 |
| CSUI_008592 | Dihydroorotate dehydrogenase (quinone), mitochondrial | Complex I | 1.018 | 2.653 | 1.450 | -0.970 |
| CSUI_006257 | Non-proton pumping type-ii nadh dehydrogenase i | Complex I | 1.076 | 1.944 | 0.562 | -2.440 |
| CSUI_001036 | Mitochondrial p5cdh | Complex I | 0.796 | 1.906 | 0.161 | -2.581 |
| CSUI_006121 | Nadh dehydrogenase (Ndh2-ii) | Complex I | 0.859 | 1.651 | -0.395 | -2.496 |
| CSUI_003030 | Flavoprotein subunit of succinate dehydrogenase | Complex II | 1.006 | 1.995 | 0.941 | -1.614 |
| CSUI_009970 | Flavoprotein subunit of succinate dehydrogenase (Fragment) | Complex II | 0.178 | 1.073 | -0.027 | -2.089 |
| CSUI_008373 | Mitochondrial processing peptidase alpha subunit | Complex III | 1.151 | 1.991 | 0.502 | -2.186 |
| CSUI_009157 | Ubiquinol-cytochrome c reductase (Fragment) | Complex III | 0.987 | 1.696 | 0.432 | -2.903 |
| CSUI_011157 | Ubiquinol-cytochrome c family reductase uqcrx qcr9-like protein | Complex III | 0.927 | 1.692 | 0.333 | -2.945 |
| CSUI_006944 | Cytochrome heme protein | Complex III | 0.842 | 1.644 | 0.255 | -1.242 |
| CSUI_011408 | Mitochondrial processing peptidase alpha subunit | Complex III | 0.781 | 1.764 | 0.195 | -3.062 |
| CSUI_002953 | Ubiquinol-cytochrome c iron-sulfur subunit | Complex III | 0.734 | 1.470 | 0.180 | -3.042 |
| CSUI_003199 | hypothetical protein | Complex IV | 1.162 | 2.114 | 0.990 | -1.846 |
| CSUI_010335 | hypothetical protein | Complex IV | 0.620 | 1.446 | 0.403 | -2.221 |
| CSUI_003996 | Cytochrome c oxidase subunit | Complex IV | 0.442 | 1.290 | 0.368 | -1.628 |
| CSUI_004801 | Nadh-ubiquinone reductase Complex 1 mlrq subunit | Complex IV | 0.369 | 1.371 | 0.243 | -2.258 |
| CSUI_009402 | Cytochrome c oxidase subunit (Fragment) | Complex IV | 0.637 | 1.520 | 0.211 | -2.623 |
| CSUI_004322 | hypothetical protein | Complex IV | 0.437 | 1.503 | 0.186 | -3.413 |
| CSUI_009393 | Cytochrome b5 family heme steroid binding domain-containing protein | Complex IV | 0.623 | 1.399 | 0.162 | -0.366 |
| CSUI_005351 | Cytochrome p450 superfamily protein | Complex IV | 0.734 | 1.572 | 0.093 | -2.278 |
| CSUI_009401 | Cytochrome c oxidase subunit (Fragment) | Complex IV | -0.576 | 0.659 | -0.122 | -2.687 |
| CSUI_009861 | hypothetical protein | Complex IV | -0.097 | 0.806 | -0.594 | -2.373 |
| CSUI_009262 | Cytochrome c oxidase subunit | Complex IV | -0.390 | 0.394 | -0.821 | -2.549 |
| CSUI_003887 | hypothetical protein | Complex V | 0.569 | 1.289 | 1.073 | -0.306 |
| CSUI_001664 | Atp synthase delta subunit protein | Complex V | 1.475 | 2.373 | 0.906 | -3.164 |
| CSUI_002556 | Inorganic diphosphatase (Fragment) | Complex V | 0.751 | 2.196 | 0.822 | -0.351 |
| CSUI_006204 | Atp synthase subunit alpha | Complex V | 1.230 | 2.104 | 0.789 | -1.954 |
| CSUI_000365 | hypothetical protein | Complex V | 1.001 | 1.902 | 0.659 | -1.920 |
| CSUI_000236 | V-type proton ATPase subunit | Complex V | 1.109 | 1.938 | 0.650 | -1.443 |
| CSUI_002965 | hypothetical protein | Complex V | 0.935 | 1.954 | 0.607 | -3.708 |
| CSUI_008670 | Transmembrane protein | Complex V | 0.764 | 1.684 | 0.544 | -3.833 |
| CSUI_005291 | hypothetical protein | Complex V | 0.974 | 1.937 | 0.494 | -2.807 |
| CSUI_008797 | hypothetical protein | Complex V | 1.063 | 2.048 | 0.460 | -3.516 |
| CSUI_004872 | ATP synthase subunit beta | Complex V | 0.711 | 1.650 | 0.238 | -2.219 |
| CSUI_006929 | Atp synthase f1 gamma subunit | Complex V | 0.529 | 1.548 | 0.221 | -3.005 |
| CSUI_006144 | Bt1 folate biopterin transporter family related | Complex V | 0.603 | 1.566 | 0.085 | -2.314 |
| CSUI_001624 | Atp synthase | Complex V | 0.799 | 1.765 | -0.150 | -2.980 |
| CSUI_008235 | Mitochondrial atp synthase epsilon | Complex V | 1.401 | 2.309 | -0.294 | -3.241 |
| CSUI_009527 | Vacuolar atp synthase subunit | Complex V | 0.509 | 1.077 | -0.640 | -2.455 |
| CSUI_002039 | Transmembrane protein | Complex V | 0.331 | 0.771 | -0.904 | -3.792 |
| CSUI_005316 | Vacuolar proton pump subunit B | Complex V | 0.572 | 1.068 | -1.877 | -2.952 |
| CSUI_006684 | H(+)-transporting two-sector ATPase | Complex V | 0.268 | 0.964 | -2.220 | -4.593 |
| CSUI_006510 | V-type proton ATPase proteolipid subunit | Complex V | -1.081 | 0.181 | -2.860 | -4.107 |
| CSUI_004411 | Cytochrome c | electron carrier | 0.754 | 1.670 | 0.389 | -2.013 |
| CSUI_000107 | Sco1 protein | cytochrome c oxidase assembly protein | 1.574 | 2.262 | 0.385 | -1.952 |

**Table S3. Proteins with putative roles in the lipid metabolism.** They are listed along with their annotation number in ToxoDB, protein name, biological function, and abundance (LogFC), in each comparison. Early merozoites (day 7, C7) versus late merozoites (day 8, C8), versus sexual stages (day 10, C10), mature sexual stages (day 12, C12) and oocysts (day 14, C14).

| **ProbeID** | **Protein name** | **Biological function** | **C 7_8** | **C 7_10** | **C 7_12** | **C 7_14** |
| --- | --- | --- | --- | --- | --- | --- |
| CSUI_004068 | Carbonyl reductase | Arachidonic acid metabolism | 0.708 | 1.740 | 0.120 | -2.002 |
| CSUI_001762 | Short chain dehydrogenase reductase family protein | fatty acid biosynthetic process | 0.261 | 0.844 | 2.481 | 0.982 |
| CSUI_010330 | Acyl carrier protein | fatty acid biosynthetic process | 1.514 | 2.756 | 2.079 | -0.972 |
| CSUI_002612 | 3-ketoacyl-(Acyl-carrier-protein) reductase | fatty acid biosynthetic process | 1.263 | 2.319 | 1.543 | -1.414 |
| CSUI_003216 | Short chain dehydrogenase reductase family protein | fatty acid biosynthetic process | -0.339 | 0.102 | 1.098 | 0.420 |
| CSUI_008840 | 3-hydroxybutyryl-dehydrogenase | fatty acid biosynthetic process | 1.929 | 2.910 | 0.914 | -1.525 |
| CSUI_000883 | acetyl- carboxylase acc1 | fatty acid biosynthetic process | 0.564 | 1.259 | 0.557 | -1.531 |
| CSUI_000890 | Beta-ketoacyl-[acyl-carrier-protein] synthase I | fatty acid biosynthetic process | 0.478 | 1.580 | 0.536 | -2.696 |
| CSUI_001389 | Enoyl-acyl carrier reductase enr | fatty acid biosynthetic process | 0.712 | 1.859 | 0.410 | -2.577 |
| CSUI_000974 | Amp-binding enzyme domain-containing protein | fatty acid biosynthetic process | 0.818 | 1.467 | -0.079 | -1.931 |
| CSUI_003297 | Acyl-coa-binding protein (ACBP) | fatty acid biosynthetic process | 0.537 | 1.367 | -0.102 | -0.716 |
| CSUI_000711 | Amp-binding enzyme domain-containing protein | fatty acid biosynthetic process | 0.877 | 1.418 | -0.421 | -2.439 |
| CSUI_010811 | Amp-binding enzyme domain-containing protein | fatty acid biosynthetic process | 0.744 | 1.802 | -0.915 | -2.627 |
| CSUI_007761 | Long-chain fatty acid ligase | fatty acid biosynthetic process | 1.651 | 2.404 | -0.983 | -1.508 |
| CSUI_004552 | Elongation of fatty acids protein | fatty acid biosynthetic process | -1.052 | -0.386 | -1.461 | -1.177 |
| CSUI_003655 | Strictosidine synthase subfamily | glycerophospholipid met | 2.862 | 3.891 | 2.835 | -0.752 |
| CSUI_009793 | Glycerophosphodiester phosphodiesterase family protein | glycerophospholipid met | 1.280 | 2.510 | 0.518 | -1.268 |
| CSUI_007585 | Phospholipase | glycerophospholipid met | 1.663 | 2.951 | 3.366 | 1.835 |
| CSUI_002440 | Acyltransferase domain-containing protein | glycerophospholipid met | 0.626 | 1.676 | 2.742 | -0.013 |
| CSUI_005022 | Phosphatidylserine decarboxylase | glycerophospholipid met | 2.059 | 3.019 | 1.617 | 0.151 |
| CSUI_006992 | Acyltransferase domain-containing protein (ATSI) | glycerophospholipid met-degra | 0.428 | 1.460 | 0.675 | -0.695 |
| CSUI_005869 | Fad-dependent glycerol-3-phosphate dehydrogenase (Fragment) | glycerophospholipid met | 0.710 | 1.345 | -0.018 | -2.279 |
| CSUI_006920 | Glycerol-3-phosphate dehydrogenase [NAD(+)] | glycerophospholipid met | 0.260 | 0.944 | -0.613 | -1.617 |
| CSUI_003666 | Glyceraldehyde-3-phosphate dehydrogenase | glycerophospholipid met | 0.629 | 1.362 | 0.164 | -1.526 |
| CSUI_002215 | Lipase | lipid metabolic process | 0.206 | 0.487 | -0.506 | -0.514 |
| CSUI_009629 | 3-oxo-5-alpha-steroid 4-dehydrogenase | lipid metabolic process | 0.213 | 1.038 | -0.541 | -3.202 |
| CSUI_002253 | Patatin family protein | lipid metabolic process | 0.443 | 1.151 | -1.238 | -3.648 |

**Table S4. Proteins with putative roles in the amino acid, nucleotide, and folate metabolism.** They are listed along with their annotation number in ToxoDB, protein name, biological function, and abundance (LogFC), in each comparison. Early merozoites (day 7, C7) versus late merozoites (day 8, C8), versus sexual stages (day 10, C10), mature sexual stages (day 12, C12) and oocysts (day 14, C14).

| **ProbeID** | **Protein name** | **biology function** | **C 7_8** | **C 7_10** | **C 7_12** | **C 7_14** |
| --- | --- | --- | --- | --- | --- | --- |
| CSUI_001049 | Aminotransferase | amino acid metabolic process | 0.365 | 1.010 | -0.180 | -1.546 |
| CSUI_004056 | Major facilitator family protein | arginine-transmembrane transport | 1.112 | 2.316 | 0.011 | -3.406 |
| CSUI_008676 | Aspartate transaminase | Asp-amino acid metabolic process | 0.528 | 1.318 | -0.288 | -3.180 |
| CSUI_006263 | Asparagine synthase (glutamine-hydrolyzing) | Asp-amino acid metabolic process | 0.580 | 0.551 | -0.406 | -1.116 |
| CSUI_003412 | Asparagine synthase (glutamine-hydrolyzing) | Asp-amino acid metabolic process | 0.908 | 1.457 | -2.282 | -2.717 |
| CSUI_001547 | Aspartate aminotransferase | Asp-amino acid metabolic process | -2.617 | -2.312 | -2.866 | -0.031 |
| CSUI_001205 | Cysteine desulfurase | folate metabolism | 1.007 | 1.940 | 0.783 | -1.566 |
| CSUI_005728 | Cysteine desulfurase selenocysteine lyase family plp dependent transferase superfamily protein | folate metabolism | 0.811 | 1.578 | 0.581 | -1.304 |
| CSUI_001304 | dihydropteroate synthase | folate metabolism | 1.329 | 1.957 | 0.058 | -2.853 |
| CSUI_010297 | Glutamate leucine phenylalanine valine dehydrogenase family protein (Fragment) | Glut-amino acid metabolic process | 1.092 | 1.675 | 0.592 | -3.321 |
| CSUI_010774 | Glutamate leucine phenylalanine valine dehydrogenase family protein (Fragment) | Glut-amino acid metabolic process | 0.763 | 1.239 | 0.042 | -0.877 |
| CSUI_010235 | Glutamate leucine phenylalanine valine dehydrogenase family protein (Fragment) | Glut-amino acid metabolic process | 1.500 | 1.967 | -0.277 | -3.824 |
| CSUI_003150 | Glutamine type i | Glut-amino acid metabolic process | 0.575 | 1.330 | -1.207 | -3.043 |
| CSUI_001515 | glycine cleavage t-protein (aminomethyl transferase) domain-containing protein | glycine catabolic process/folate metabolims | 1.912 | 3.684 | 0.406 | 0.239 |
| CSUI_002776 | Aspartate-semialdehyde dehydrogenase | Lysine/threonine-amino acid metabolic process | 1.085 | 2.093 | -1.047 | -1.129 |
| CSUI_001859 | dihydrodipicolinate synthase | Lysine-amino acid metabolic process | 0.090 | 0.429 | 0.988 | 1.037 |
| CSUI_006501 | Carbon-nitrogen family protein | nitrogen compound metabolic process | 0.754 | 1.760 | 0.039 | -1.358 |
| CSUI_001107 | Proline dehydrogenase | Proline catabolic process | 0.646 | 1.177 | -0.029 | -0.809 |
| CSUI_000255 | Pyrroline-5-carboxylate reductase | Proline-amino acid metabolic process | 1.416 | 2.784 | 0.041 | -2.092 |
| CSUI_007993 | D-3-phosphoglycerate dehydrogenase | serine-amino acid metabolic process | 0.847 | 1.515 | 0.560 | -0.620 |
| CSUI_010078 | Serine hydroxymethyltransferase | serine-amino acid metabolic process/folate metabolism | 0.633 | 0.933 | -0.276 | -0.595 |
| CSUI_009280 | phosphoserine aminotransferase | serine-amino acid metabolic process | -0.310 | 1.209 | -1.127 | -2.178 |
| CSUI_011414 | tryptophan synthase subunit beta | tryptophan-amino acid metabolic process | 0.434 | 0.226 | -1.340 | -3.363 |
| CSUI_006182 | Major facilitator family protein | tyrosine-transmembrane transport | 0.962 | 1.575 | 0.219 | -2.094 |
| CSUI_009980 | Phosphoribosylpyrophosphate synthetase | purine | 0.625 | 1.608 | 0.549 | -1.867 |
| CSUI_000469 | Phosphoglucomutase parafusin related protein | purine | -0.129 | 0.669 | -0.893 | -2.667 |
| CSUI_010965 | Nucleoside diphosphate kinase | purine | -0.003 | 0.836 | -1.219 | -3.981 |
| CSUI_008798 | Transmembrane protein | purine | 0.850 | 1.014 | -1.455 | -3.032 |
| CSUI_006926 | Glutamine glutamic acid rich protein | purine | -0.462 | 0.272 | -3.671 | -4.106 |
| CSUI_006138 | Adenylate kinase | purine | 0.822 | 1.812 | 0.615 | -2.569 |
| CSUI_006184 | bifunctional gmp synthase glutamine amidotransferase protein | purine | -0.129 | 0.072 | -0.317 | -1.582 |
| CSUI_002697 | Hypoxanthine-xanthine-guanine phosphoribosyl transferase hxgprt (Fragment) | purine | 0.486 | 1.316 | -0.631 | -3.957 |
| CSUI_008968 | inosine-5 -monophosphate | purine | 0.514 | 1.047 | -2.631 | -3.721 |
| CSUI_001313 | Orotate phosphoribosyltransferase | pyrimidine | 1.006 | 1.915 | -0.372 | -1.110 |
| CSUI_007385 | Purine nucleoside phosphorylase | pyrimidine | 0.650 | 1.729 | -0.650 | -1.316 |
| CSUI_005053 | Deoxyuridine 5'-triphosphate nucleotidohydrolase | pyrimidine | 0.193 | 1.235 | -0.881 | -3.592 |
| CSUI_002733 | Carbamoylphosphate synthetase | pyrimidine | 0.517 | 0.989 | -0.981 | -1.431 |
| CSUI_006464 | Bifunctional dihydrofolate reductase-thymidylate synthase | pyrimidine /folate metabolism | 0.478 | 1.130 | -1.258 | -3.039 |
| CSUI_001256 | Uracil phosphoribosyltransferase | pyrimidine | 0.971 | 2.081 | -2.189 | -3.245 |
| CSUI_000935 | CTP synthase | pyrimidine | -2.366 | -2.070 | -2.907 | -0.820 |
| CSUI_008592 | Dihydroorotate dehydrogenase (quinone), mitochondrial | pyrimidine/folate metabolism | 1.018 | 2.653 | 1.450 | -0.970 |

**Table S5. Proteins with putative roles in processing of genetic information.** They are listed along with their annotation number in ToxoDB, protein name, biological function, and abundance (LogFC), in each comparison. Early merozoites (day 7, C7) versus late merozoites (day 8, C8), versus sexual stages (day 10, C10), mature sexual stages (day 12, C12) and oocysts (day 14, C14).

| **ProbeID** | **Protein name** | **Function** | **C 7_8** | **C 7_10** | **C 7_12** | **C 7_14** |
| --- | --- | --- | --- | --- | --- | --- |
| CSUI_010299 | Cell-cycle-associated protein kinase gsk | cell cycle regulation | 0.130 | 0.501 | 0.191 | -1.805 |
| CSUI_002626 | Regulator of chromosome condensation rcc1 | cell cycle regulation | 0.667 | 1.196 | -0.159 | -0.577 |
| CSUI_001136 | Cell division protein cdc48cy | cell cycle regulation | 0.170 | 0.665 | -1.374 | -2.668 |
| CSUI_002060 | Histone H4 | Chromatin complex | 2.027 | 2.915 | 2.968 | 1.199 |
| CSUI_002076 | Histone H3 | Chromatin complex | 1.996 | 2.956 | 2.893 | 1.131 |
| CSUI_008436 | Protein-serine/threonine kinase | Chromatin complex | 2.176 | 4.096 | 2.851 | 0.847 |
| CSUI_003118 | Methyltransferase | Chromatin complex | 0.905 | 2.578 | 2.575 | 0.192 |
| CSUI_007420 | RuvB-like helicase | Chromatin complex | 1.245 | 2.313 | 1.634 | 1.236 |
| CSUI_007597 | histone H2AX | Chromatin complex | 1.346 | 2.241 | 1.457 | -0.956 |
| CSUI_007088 | RuvB family 2 RuvB family 2 protein | Chromatin complex | 1.045 | 1.926 | 1.427 | 0.416 |
| CSUI_002225 | Histone H2Ba | Chromatin complex | 0.883 | 1.707 | 1.376 | -0.203 |
| CSUI_005845 | Protein-serine/threonine kinase | Chromatin complex | 0.718 | 1.573 | 0.913 | -3.152 |
| CSUI_010473 | Histone H2BZ | Chromatin complex | 0.928 | 1.835 | 0.887 | -1.022 |
| CSUI_000266 | Dna-binding protein | Chromatin complex | 0.934 | 1.364 | 0.328 | -1.768 |
| CSUI_001093 | histone H2AZ | Chromatin complex | 0.197 | 0.895 | -0.193 | -1.650 |
| CSUI_002075 | histone H2AZ | Chromatin complex | 0.074 | 1.509 | -0.615 | -1.373 |
| CSUI_011319 | Cold-shock dna-binding domain-containing protein | DNA binding | -0.128 | -0.040 | -3.382 | -6.004 |
| CSUI_008295 | S1 p1nuclease | DNA catabolic process | 0.458 | 1.400 | -1.241 | -3.909 |
| CSUI_001214 | Replication factor c subunit | DNA replication | 0.201 | 0.663 | 1.001 | 0.535 |
| CSUI_003256 | Nuclear factor nf7 | DNA replication | 0.276 | 0.411 | -0.460 | -1.375 |
| CSUI_004913 | dna replication licensing factor mcm5 | DNA replication | 1.430 | 1.717 | -0.646 | -0.607 |
| CSUI_002598 | DNA replication licensing factor MCM2 | DNA replication | 1.234 | 1.334 | -0.880 | -0.648 |
| CSUI_009120 | dna replication licensing factor | DNA replication | 0.805 | 1.340 | -1.020 | -1.831 |
| CSUI_009276 | Nuclear factor nf3 | DNA replication | 0.536 | 1.295 | -1.079 | -3.417 |
| CSUI_007201 | DNA replication licensing factor | DNA replication | 0.726 | 0.158 | -2.215 | -2.194 |
| CSUI_002392 | Activator 1 36 | DNA replication | 0.233 | 0.412 | -2.524 | -2.460 |
| CSUI_008445 | Nuclear transport factor 2 domain-containing protein | DNA transport | 1.004 | 1.708 | -0.641 | -1.610 |
| CSUI_011028 | Mitotic checkpoint protein (Fragment) | mitosis regulator | 1.116 | 1.978 | -0.147 | -1.964 |
| CSUI_001174 | Cmcg kinase (Incomplete catalytic triad) | protein phosphorylation | 0.827 | 1.692 | 1.083 | -1.268 |
| CSUI_010999 | Camp-dependent protein kinase regulatory | regulation of protein phosphorylation | -0.086 | 0.143 | 0.917 | 0.276 |
| CSUI_005479 | g2 protein | regulator of mitosis | 0.980 | 1.697 | -0.769 | -4.617 |
| CSUI_009209 | Ma3 domain-containing protein | negative regulation of transcription, DNA-templated | 0.396 | 0.564 | -0.620 | -0.852 |
| CSUI_011582 | Corepressor complex crc230 | regulation of Transcription | 0.883 | 1.710 | 0.230 | -0.092 |
| CSUI_008211 | Corepressor complex crc230 | regulation of Transcription | 0.555 | 1.059 | -0.755 | -1.197 |
| CSUI_011258 | Corepressor complex crc230 | regulation of Transcription | 1.056 | 2.029 | -0.967 | -2.606 |
| CSUI_005300 | Gcn1 | regulation of Translation | 0.640 | 1.566 | -0.957 | -1.900 |
| CSUI_005790 | H/ACA ribonucleoprotein complex subunit | ribosome biogenesis | 2.540 | 2.716 | 1.728 | -2.086 |
| CSUI_003543 | Fibrillarin | ribosome biogenesis | 1.492 | 2.642 | 0.271 | -4.741 |
| CSUI_008852 | Nucleolar protein | ribosome biogenesis | 0.829 | 1.689 | -0.297 | -3.165 |
| CSUI_002320 | atpase protein | ribosome biogenesis | 0.600 | 1.776 | -0.506 | -2.488 |
| CSUI_008694 | u3 small nucleolar rna-associated protein 10 | ribosome biogenesis | 1.088 | 2.134 | -0.899 | -1.492 |
| CSUI_001559 | Cmgc ck2 family | ribosome biogenesis | 1.216 | 1.751 | -1.690 | -3.250 |
| CSUI_002783 | Nucleolar protein | ribosome biogenesis | 0.067 | 1.287 | -1.867 | -3.279 |
| CSUI_000479 | Rap domain-containing protein | RNA binding | 1.162 | 2.697 | 2.724 | 1.081 |
| CSUI_006809 | dead deah box helicase domain-containing protein | RNA binding | 1.542 | 2.533 | 0.692 | -1.516 |
| CSUI_002933 | Rna-binding protein | RNA binding | 1.181 | 2.148 | -0.277 | -2.619 |
| CSUI_003538 | Kh domain-containing | RNA binding | 0.249 | 0.544 | -0.473 | -0.215 |
| CSUI_000246 | Domain k-type rna binding proteins family protein | RNA binding | 0.727 | 1.630 | -0.503 | -2.723 |
| CSUI_002208 | Mif4g domain-containing | RNA binding | 1.226 | 2.078 | -1.063 | -3.895 |
| CSUI_000270 | Rna-binding protein nova-1 | RNA binding | 0.966 | 1.861 | -1.134 | -4.300 |
| CSUI_001704 | Kh domain-containing | RNA binding | 0.059 | 0.448 | -1.482 | -2.758 |
| CSUI_002462 | Hyaluronan mrna-binding family protein | RNA binding | 0.187 | 0.801 | -2.029 | -5.232 |
| CSUI_007168 | Rrm domain-containing | RNA binding | -1.657 | -0.820 | -2.253 | -3.085 |
| CSUI_000297 | ribosomel rna large subunit methyltransferase j protein | RNA methylation | -1.472 | 0.072 | 0.048 | 0.841 |
| CSUI_010472 | dna chr wayne state university expressed family protein | RNA processing | 2.380 | 2.090 | 1.207 | -0.311 |
| CSUI_009914 | Nol1 nop2 sun family protein | RNA processing | 0.049 | 0.715 | -0.091 | -0.367 |
| CSUI_006579 | Proliferation-associated protein 2g4 | RNA-binding | 0.418 | 0.962 | -1.304 | -3.295 |
| CSUI_010101 | nac domain-containing protein | Transcription activator | -0.031 | 0.576 | -1.366 | -4.937 |
| CSUI_002472 | Nac domain-containing protein | Transcription activator | -0.112 | 1.043 | -2.293 | -5.817 |
| CSUI_010036 | Splicing factor | Transcription-factor | 1.019 | 1.969 | 0.352 | -1.401 |
| CSUI_001915 | Splicing factor 3b subunit | Transcription-factor | 0.848 | 1.697 | -0.230 | -1.433 |
| CSUI_006403 | Splicing factor subunit | Transcription-factor | 0.678 | 1.408 | -0.324 | -1.869 |
| CSUI_001815 | Splicing factor u2af family auxilary factor large rrm domain-containing protein | Transcription-factor | -0.448 | 0.085 | -2.409 | -3.606 |
| CSUI_004213 | Dead deah box atp-dependent rna helicase | Transcription-spliceosome | 2.246 | 2.450 | 1.795 | -0.722 |
| CSUI_003136 | Splicing factor u2af protein | Transcription-spliceosome | 1.494 | 2.136 | 1.480 | -0.090 |
| CSUI_008170 | Elongation factor Tu | Transcription-spliceosome | 0.762 | 1.775 | 0.355 | -2.473 |
| CSUI_003371 | U5 snrnp-associated 102 kDa | Transcription-spliceosome | -0.778 | -0.629 | 0.260 | 1.497 |
| CSUI_008657 | Rna recognition motif-containing protein | Transcription-spliceosome | 0.935 | 1.721 | 0.131 | -2.177 |
| CSUI_006010 | U6 snrna-associated sm family protein lsm6 | Transcription-spliceosome | 0.980 | 2.102 | 0.033 | -1.206 |
| CSUI_006393 | Atp-dependent rna helicase | Transcription-spliceosome | 0.823 | 2.437 | 0.033 | -2.187 |
| CSUI_009669 | Dead (Asp-glu-ala-asp) box polypeptide 17 | Transcription-spliceosome | 0.531 | 1.173 | 0.014 | -1.125 |
| CSUI_002827 | Elongation factor p | Transcription-spliceosome | 1.150 | 2.521 | -0.097 | -2.135 |
| CSUI_008216 | Dead (Asp-glu-ala-asp) box polypeptide ddx3x | Transcription-spliceosome | 0.350 | 0.850 | -0.280 | -0.681 |
| CSUI_004211 | Atp-dependent rna | Transcription-spliceosome | 0.161 | 0.550 | -0.354 | -0.748 |
| CSUI_010273 | Dead deah box helicase | Transcription-spliceosome | 0.467 | 0.713 | -0.359 | -1.084 |
| CSUI_009858 | Rna recognition motif-containing protein | Transcription-spliceosome | 0.750 | 1.320 | -0.419 | -1.294 |
| CSUI_001330 | Rna recognition motif-containing protein | Transcription-spliceosome | 0.587 | 1.303 | -0.459 | -1.939 |
| CSUI_001711 | Sec63 domain-containing dead deah box | Transcription-spliceosome | 1.190 | 1.367 | -0.479 | -2.209 |
| CSUI_011386 | Rna recognition motif-containing protein (Fragment) | Transcription-spliceosome | 0.712 | 1.391 | -0.533 | -2.338 |
| CSUI_003756 | Ethylene-responsive rna helicase | Transcription-spliceosome | 0.991 | 1.867 | -0.573 | -3.325 |
| CSUI_003116 | Rna recognition motif-containing protein | Transcription-spliceosome | 0.408 | 0.911 | -0.731 | -0.313 |
| CSUI_001097 | Elongation factor 1-gamma | Transcription-spliceosome | 0.379 | 1.044 | -0.871 | -2.678 |
| CSUI_001080 | Dead (Asp-glu-ala-asp) box polypeptide ddx39 | Transcription-spliceosome | 0.180 | 1.268 | -0.914 | -2.405 |
| CSUI_000967 | Rna recognition motif-containing protein | Transcription-spliceosome | 0.371 | 0.944 | -0.999 | -3.954 |
| CSUI_010060 | Rna recognition motif-containing protein | Transcription-spliceosome | 0.685 | 1.136 | -1.036 | -4.272 |
| CSUI_007172 | nhp2-like protein 1 | Transcription-spliceosome | 1.110 | 1.881 | -1.113 | -2.289 |
| CSUI_002779 | Elongation factor | Transcription-spliceosome | 0.335 | 0.999 | -1.185 | -3.182 |
| CSUI_001242 | Rna recognition motif-containing protein | Transcription-spliceosome | -0.085 | 0.847 | -1.236 | -1.284 |
| CSUI_010438 | Rna recognition motif-containing protein (Fragment) | Transcription-spliceosome | 0.167 | 0.936 | -1.244 | -2.385 |
| CSUI_005037 | Rna recognition motif-containing | Transcription-spliceosome | 0.149 | 0.859 | -1.389 | -3.885 |
| CSUI_003474 | Elongation factor 1-alpha | Transcription-spliceosome | 0.243 | 0.741 | -1.400 | -2.744 |
| CSUI_002405 | Small nuclear ribonucleoprotein polypeptide a | Transcription-spliceosome | 0.323 | 0.778 | -1.433 | -2.047 |
| CSUI_002405 | Small nuclear ribonucleoprotein polypeptide a | Transcription-spliceosome | 0.323 | 0.778 | -1.433 | -2.047 |
| CSUI_010599 | Small nuclear ribonucleoprotein Sm D2 | Transcription-spliceosome | -0.046 | 0.850 | -1.709 | -2.120 |
| CSUI_010368 | Rna recognition motif-containing protein | Transcription-spliceosome | 0.278 | 0.849 | -1.832 | -3.643 |
| CSUI_001835 | Rna recognition motif-containing protein | Transcription-spliceosome | -0.012 | 0.735 | -1.849 | -3.619 |
| CSUI_003926 | Small nuclear ribonucleoprotein Sm D3 | Transcription-spliceosome | -0.572 | 0.659 | -2.166 | -1.524 |
| CSUI_005841 | Rna recognition motif-containing protein | Transcription-spliceosome | -0.248 | 0.345 | -2.175 | -2.002 |
| CSUI_009830 | Dna rna-binding protein alba | Transcription-spliceosome | 0.359 | 0.658 | -2.938 | -6.758 |
| CSUI_006011 | FACT complex subunit SSRP1 | Translation elongation factor activity | 0.352 | 0.740 | 0.225 | -1.679 |
| CSUI_006331 | FACT complex subunit | Translation elongation factor activity | 0.122 | 1.221 | -0.717 | -2.573 |
| CSUI_001466 | Ef-1 guanine nucleotide exchange domain-containing protein | Translation elongation factor activity | 0.486 | 1.093 | -0.849 | -2.641 |
| CSUI_007674 | Translation initiation factor if-2 | Translation elongation factor activity | 0.021 | 0.349 | -2.516 | -2.376 |
| CSUI_002646 | Translationally-controlled tumor | Translation elongation factor activity | 0.486 | 1.342 | -2.573 | -4.707 |
| CSUI_001554 | Eukaryotic initiation factor- epsilon | Translation initiation factor activity | 2.742 | 3.033 | 2.638 | 0.336 |
| CSUI_007043 | Eukaryotic Translation initiation factor | Translation initiation factor activity | 1.982 | 2.556 | 1.679 | -1.581 |
| CSUI_008653 | Eukaryotic initiation factor-3 subunit | Translation initiation factor activity | 1.955 | 1.872 | 1.425 | -1.583 |
| CSUI_002764 | Eukaryotic initiation factor 4a-iii | Translation initiation factor activity | 0.736 | 1.698 | 0.410 | -2.100 |
| CSUI_000248 | Eukaryotic Translation initiation factor 2 gamma | Translation initiation factor activity | 0.280 | 0.986 | -0.142 | -1.036 |
| CSUI_003128 | Eukaryotic Translation initiation factor 3 subunit C | Translation initiation factor activity | 0.731 | 1.435 | -0.220 | -1.478 |
| CSUI_001861 | Eukaryotic Translation initiation factor 3 subunit | Translation initiation factor activity | 0.416 | 1.200 | -0.291 | -1.857 |
| CSUI_004102 | Eukaryotic Translation initiation factor 3 subunit I | Translation initiation factor activity | 0.630 | 1.147 | -0.382 | -1.239 |
| CSUI_007946 | Eukaryotic peptide chain release factor subunit 1 | Translation initiation factor activity | 0.353 | 1.421 | -0.595 | -2.436 |
| CSUI_010474 | Eukaryotic initiation factor 4a | Translation initiation factor activity | 0.390 | 1.030 | -0.951 | -2.284 |
| CSUI_006602 | Eukaryotic Translation initiation factor 2 alpha | Translation initiation factor activity | -0.838 | -0.065 | -1.046 | -1.521 |
| CSUI_010564 | Eukaryotic Translation initiation factor 3 subunit 6 interacting protein (Fragment) | Translation initiation factor activity | 0.567 | 1.079 | -1.082 | -3.403 |
| CSUI_001489 | Eukaryotic Translation initiation factor 3 subunit | Translation initiation factor activity | 0.260 | 0.822 | -1.114 | -3.235 |
| CSUI_001712 | Eukaryotic initiation factor-4e | Translation initiation factor activity | 0.600 | 1.148 | -1.151 | -2.136 |
| CSUI_000550 | Eukaryotic Translation initiation factor 3 subunit B | Translation initiation factor activity | 0.355 | 0.701 | -1.407 | -1.985 |
| CSUI_000560 | Eukaryotic Translation initiation factor 3 30 kDa subunit | Translation initiation factor activity | 0.279 | 0.845 | -1.562 | -2.707 |
| CSUI_008378 | Eukaryotic Translation initiation factor 2 alpha | Translation initiation factor activity | 0.467 | 1.298 | -1.635 | -3.556 |
| CSUI_003300 | Eukaryotic Translation initiation factor 3 subunit H | Translation initiation factor activity | 0.370 | 0.858 | -1.717 | -4.231 |
| CSUI_011531 | Eukaryotic Translation initiation factor 3 subunit 10 | Translation initiation factor activity | 0.549 | 1.062 | -1.732 | -2.472 |
| CSUI_002058 | Serine/threonine-protein phosphatase | Translation-mRNA surveillance | 0.862 | 1.359 | 0.304 | -0.540 |
| CSUI_005939 | Polyadenylate-binding protein | Translation-mRNA surveillance | 0.561 | 1.056 | -0.526 | -2.195 |
| CSUI_007109 | Serine/threonine-protein phosphatase | Translation-mRNA surveillance | 0.985 | 1.408 | -0.805 | -2.675 |
| CSUI_009475 | Serine-threonine phosophatase 2c | Translation-mRNA surveillance | 0.279 | 0.985 | -0.973 | -3.138 |
| CSUI_004047 | Serine/threonine-protein phosphatase | Translation-mRNA surveillance | -1.094 | 0.275 | -1.471 | -3.409 |
| CSUI_011136 | 60S ribosomel protein L7a | Translation-ribosome | 1.450 | 2.626 | 1.339 | 0.156 |
| CSUI_000820 | ribosomel l1p l10e family protein | Translation-ribosome | 2.015 | 3.676 | 0.710 | -1.461 |
| CSUI_011029 | 40S ribosomel protein S8 | Translation-ribosome | 0.886 | 1.727 | 0.705 | -1.238 |
| CSUI_004271 | 40s ribosomel protein | Translation-ribosome | 1.385 | 2.518 | 0.581 | -2.468 |
| CSUI_003987 | ribosomel protein | Translation-ribosome | 1.253 | 2.087 | 0.481 | -2.673 |
| CSUI_006991 | ribosomel protein rpl17 | Translation-ribosome | 1.638 | 2.352 | 0.412 | -1.592 |
| CSUI_010067 | 60s ribosomel protein | Translation-ribosome | 1.386 | 2.194 | 0.382 | -2.420 |
| CSUI_001094 | ribosomel protein rpl37a | Translation-ribosome | 1.324 | 2.075 | 0.364 | -2.954 |
| CSUI_005111 | 60s ribosomel protein | Translation-ribosome | 1.840 | 2.366 | 0.347 | -3.777 |
| CSUI_007653 | 60s ribosomel protein | Translation-ribosome | 1.140 | 1.948 | 0.338 | -2.291 |
| CSUI_005909 | ribosomel protein rpl11 | Translation-ribosome | 1.243 | 1.986 | 0.315 | 0.141 |
| CSUI_001009 | 60s ribosomel protein l23 | Translation-ribosome | 1.181 | 1.854 | 0.256 | -2.450 |
| CSUI_008929 | 60s ribosomel protein l18 | Translation-ribosome | 1.394 | 2.143 | 0.250 | -1.710 |
| CSUI_003178 | ribosomel protein rpl35 | Translation-ribosome | 1.861 | 2.569 | 0.148 | -2.597 |
| CSUI_002139 | 60S ribosomel protein L13 | Translation-ribosome | 0.821 | 1.663 | 0.131 | -0.138 |
| CSUI_006983 | 40s ribosomel protein | Translation-ribosome | 0.555 | 1.139 | 0.130 | 0.617 |
| CSUI_004744 | 40S ribosomel protein S24 | Translation-ribosome | 1.133 | 1.880 | 0.128 | -1.957 |
| CSUI_008234 | 60s ribosomel protein | Translation-ribosome | -0.192 | 0.489 | 0.088 | -1.065 |
| CSUI_007163 | 60s ribosomel protein l33 | Translation-ribosome | 0.871 | 1.785 | 0.023 | -2.242 |
| CSUI_002593 | 40S ribosomel protein S26 | Translation-ribosome | 1.131 | 2.050 | 0.009 | -1.616 |
| CSUI_002973 | 40s ribosomel protein s23 | Translation-ribosome | 1.072 | 1.714 | -0.078 | -2.055 |
| CSUI_001860 | 40S ribosomel protein S6 | Translation-ribosome | 0.937 | 1.928 | -0.104 | -2.798 |
| CSUI_004055 | ribosomel protein rpl5 | Translation-ribosome | 0.944 | 1.789 | -0.112 | -2.221 |
| CSUI_011035 | 60s ribosomel protein l21-1-like | Translation-ribosome | 0.057 | 0.659 | -0.220 | -2.156 |
| CSUI_003726 | 40s ribosomel protein | Translation-ribosome | 0.910 | 1.610 | -0.246 | -2.583 |
| CSUI_002138 | 40s ribosomel protein s16 | Translation-ribosome | 0.655 | 1.078 | -0.297 | -1.445 |
| CSUI_002982 | 60s ribosomel protein | Translation-ribosome | 1.038 | 1.869 | -0.300 | -2.396 |
| CSUI_004257 | 40S ribosomel protein S4 | Translation-ribosome | 1.037 | 1.707 | -0.314 | -2.290 |
| CSUI_008000 | 60s ribosomel protein | Translation-ribosome | 0.688 | 1.569 | -0.315 | -1.916 |
| CSUI_002243 | 40S ribosomel protein S3a | Translation-ribosome | 0.839 | 1.455 | -0.327 | -2.237 |
| CSUI_001938 | 40s ribosomel protein | Translation-ribosome | 0.936 | 1.490 | -0.330 | -2.656 |
| CSUI_009020 | ribosomel protein rpl26 | Translation-ribosome | 1.317 | 2.219 | -0.330 | -2.929 |
| CSUI_009235 | 60S ribosomel protein L36 | Translation-ribosome | -0.854 | -1.174 | -0.385 | 0.390 |
| CSUI_009318 | 60s ribosomel protein | Translation-ribosome | 0.553 | 1.121 | -0.392 | -0.696 |
| CSUI_000724 | ribosomel protein rpl34 | Translation-ribosome | 1.417 | 2.332 | -0.444 | -2.946 |
| CSUI_004226 | ribosomel protein rps13 | Translation-ribosome | 1.087 | 1.929 | -0.481 | -3.628 |
| CSUI_006651 | 60s ribosomel protein | Translation-ribosome | 1.113 | 1.856 | -0.489 | -3.544 |
| CSUI_000055 | 60S ribosomel protein L18a | Translation-ribosome | 0.789 | 1.411 | -0.491 | -1.996 |
| CSUI_006302 | ribosomel protein rpl4 | Translation-ribosome | 1.035 | 1.716 | -0.504 | -2.570 |
| CSUI_001658 | 60s ribosomel protein | Translation-ribosome | 0.691 | 1.218 | -0.575 | -2.543 |
| CSUI_002199 | 60s ribosomel protein | Translation-ribosome | 0.654 | 1.223 | -0.599 | -3.084 |
| CSUI_010375 | 40s ribosomel protein s15a | Translation-ribosome | 0.586 | 1.174 | -0.620 | -2.423 |
| CSUI_000329 | ribosomel protein | Translation-ribosome | 0.788 | 1.384 | -0.661 | -2.532 |
| CSUI_003994 | 60s ribosomel protein l27a | Translation-ribosome | 0.899 | 1.313 | -0.671 | -2.686 |
| CSUI_005897 | 40S ribosomel protein S7 | Translation-ribosome | 0.308 | 0.957 | -0.692 | -2.698 |
| CSUI_005227 | 60s ribosomel protein | Translation-ribosome | 1.245 | 1.816 | -0.695 | -3.071 |
| CSUI_004990 | ribosomel protein rps14 | Translation-ribosome | 0.738 | 1.238 | -0.748 | -2.559 |
| CSUI_003522 | ribosomel protein rpl23a | Translation-ribosome | 0.550 | 1.183 | -0.767 | -3.206 |
| CSUI_003976 | 60s ribosomel protein | Translation-ribosome | 0.623 | 1.390 | -0.853 | -2.836 |
| CSUI_006793 | ribosomel protein L15 | Translation-ribosome | 1.058 | 1.572 | -0.866 | -3.483 |
| CSUI_001821 | ribosomel protein rps3 | Translation-ribosome | 0.217 | 0.671 | -0.912 | -2.646 |
| CSUI_000047 | 40s ribosomel protein | Translation-ribosome | 0.788 | 1.204 | -0.930 | -2.769 |
| CSUI_006290 | 60s ribosomel protein | Translation-ribosome | 0.831 | 1.423 | -1.003 | -2.141 |
| CSUI_009956 | 40S ribosomel protein SA | Translation-ribosome | 0.628 | 1.100 | -1.015 | -3.099 |
| CSUI_010165 | 40s ribosomel protein | Translation-ribosome | 0.413 | 0.913 | -1.149 | -3.535 |
| CSUI_009921 | 40s ribosomel protein s27 | Translation-ribosome | 0.625 | 1.160 | -1.321 | -3.224 |
| CSUI_004743 | 60s ribosomal protein | Translation-ribosome | 0.256 | 0.882 | -1.439 | -4.047 |
| CSUI_005785 | 40s ribosomel protein | Translation-ribosome | 0.357 | 0.995 | -1.527 | -3.173 |
| CSUI_001950 | 60s acidic ribosomel protein | Translation-ribosome | 0.492 | 1.076 | -1.542 | -3.622 |
| CSUI_003108 | 60s ribosomel protein | Translation-ribosome | 0.455 | 0.823 | -1.625 | -4.543 |
| CSUI_005247 | 60S acidic ribosomel protein P0 | Translation-ribosome | -0.347 | 0.294 | -2.015 | -3.886 |
| CSUI_000278 | Lysine--tRNA ligase | Translation-tRNA aminoacylation | 1.062 | 1.164 | 2.007 | 1.081 |
| CSUI_001201 | peptidyl-trna hydrolase | Translation-tRNA aminoacylation | 2.706 | 2.856 | 1.408 | 1.013 |
| CSUI_004846 | arginyl-trna synthetase | Translation-tRNA aminoacylation | 0.190 | 0.679 | 1.083 | 1.294 |
| CSUI_001493 | Methionyl-trna synthetase | Translation-tRNA aminoacylation | -1.816 | -1.060 | -0.251 | -0.466 |
| CSUI_002129 | Threonyl-tRNA synthetase | Translation-tRNA aminoacylation | 0.286 | 0.849 | -0.309 | -0.732 |
| CSUI_002958 | histidyl-trna synthetase | Translation-tRNA aminoacylation | 0.674 | 1.149 | -0.500 | -4.808 |
| CSUI_007580 | cytosolic trna-ala synthetase | Translation-tRNA aminoacylation | 0.728 | 1.356 | -0.506 | -2.584 |
| CSUI_000954 | Aspartate--tRNA ligase | Translation-tRNA aminoacylation | 0.373 | 0.743 | -0.760 | -1.535 |
| CSUI_005205 | Diadenosine tetraphosphate synthetase | Translation-tRNA aminoacylation | 0.370 | 0.689 | -0.825 | -1.237 |
| CSUI_002675 | Glutamyl-tRNA synthetase | Translation-tRNA aminoacylation | 1.439 | 2.040 | -0.825 | -2.242 |
| CSUI_002645 | Seryl-tRNA synthetase | Translation-tRNA aminoacylation | 0.552 | 1.130 | -0.837 | -4.222 |
| CSUI_001463 | Phenylalanyl-trna synthetase beta | Translation-tRNA aminoacylation | 0.926 | 2.603 | -0.949 | -1.956 |
| CSUI_003443 | Arginyl-trna synthetase family protein (Fragment) | Translation-tRNA aminoacylation | 0.207 | 0.648 | -1.161 | -0.195 |
| CSUI_003462 | Valyl-tRNA synthetase | Translation-tRNA aminoacylation | 0.457 | 0.519 | -1.228 | -1.750 |
| CSUI_005328 | Asparagine--tRNA ligase | Translation-tRNA aminoacylation | 0.670 | 1.238 | -1.254 | -3.774 |
| CSUI_001385 | Trna synthetases class i family protein | Translation-tRNA aminoacylation | -0.388 | 1.865 | -1.334 | -2.928 |
| CSUI_008164 | Tryptophanyl-tRNA synthetase | Translation-tRNA aminoacylation | -0.145 | 0.209 | -1.509 | -2.039 |
| CSUI_010261 | Glutamine--tRNA ligase | Translation-tRNA aminoacylation | 0.236 | 1.187 | -1.527 | -4.911 |
| CSUI_001735 | isoleucyl-trna synthetase family protein | Translation-tRNA aminoacylation | -0.679 | -0.324 | -1.651 | -2.185 |
| CSUI_000424 | Valyl-tRNA synthetase | Translation-tRNA aminoacylation | 0.157 | 0.950 | -1.713 | -2.370 |
| CSUI_000209 | Leucyl-tRNA synthetase | Translation-tRNA aminoacylation | -2.070 | -2.168 | -1.901 | 0.533 |
| CSUI_003005 | isoleucyl-trna synthetase family protein | Translation-tRNA aminoacylation | -0.741 | -0.073 | -2.276 | -2.387 |

**Table S6. Proteins with putative roles in the Protein folding, sorting and degradation.** They are listed along with their annotation number in ToxoDB, protein name, biological function, and abundance (LogFC), in each comparison. Early merozoites (day 7, C7) versus late merozoites (day 8, C8), versus sexual stages (day 10, C10), mature sexual stages (day 12, C12) and oocysts (day 14, C14).

| **ProbeID** | **Protein name** | **biology function** | **C 7_8** | **C 7_10** | **C 7_12** | **C 7_14** |
| --- | --- | --- | --- | --- | --- | --- |
| CSUI_001705 | Ankyrin repeat-containing protein | protein binding | 1.058 | 1.134 | 1.639 | 0.321 |
| CSUI_000578 | Ankyrin repeat-containing protein | protein binding | 0.625 | 2.289 | -0.736 | -1.480 |
| CSUI_002156 | anonymous antigen-1 | protein binding | 0.385 | 0.531 | -1.512 | -4.555 |
| CSUI_000142 | anonymous antigen-1 | protein binding | -0.088 | 0.155 | -3.209 | -4.300 |
| CSUI_007073 | Glutathione s-n-terminal domain containing protein | protein binding | -0.017 | 0.871 | -0.357 | -1.495 |
| CSUI_001078 | Guanine nucleotide-binding protein | protein binding | 0.654 | 0.903 | -0.338 | -1.379 |
| CSUI_005783 | Gyf domain-containing protein | protein binding | 1.707 | 3.356 | 2.352 | 0.822 |
| CSUI_004896 | Inhibitor-1 of protein phosphatase type 2a | protein binding | -0.431 | 0.793 | -2.032 | -2.076 |
| CSUI_002013 | Kazal-type serine protease inhibitor domain-containing protein | protein binding | -0.127 | -0.018 | -3.435 | -3.913 |
| CSUI_005478 | Leucine rich repeat protein | protein binding | 1.325 | 1.909 | 0.010 | -1.428 |
| CSUI_009737 | Leucine rich repeat-containing protein | protein binding | 0.533 | 0.709 | -1.968 | -2.183 |
| CSUI_000267 | Pci domain-containing protein | protein binding | 0.692 | 1.560 | -0.581 | -2.043 |
| CSUI_003961 | Pci domain-containing protein (Fragment) | protein binding | 0.977 | 1.608 | -0.669 | -0.685 |
| CSUI_002555 | Peptidylprolyl isomerase | protein binding | 0.303 | 0.920 | -1.153 | -3.261 |
| CSUI_006288 | Sortilin | protein binding | 0.425 | 0.759 | -0.435 | -1.483 |
| CSUI_002735 | 10 kDa | protein folding | 0.839 | 1.618 | 0.302 | -4.199 |
| CSUI_000029 | Chaperonin | protein folding | 0.670 | 1.639 | 0.668 | -0.810 |
| CSUI_000320 | Chaperonin | protein folding | 0.098 | 1.113 | 0.098 | -1.510 |
| CSUI_005049 | Co-chaperone protein | protein folding | 0.672 | 1.619 | 0.026 | -2.538 |
| CSUI_000453 | cyclophilin precursor | protein folding | 0.887 | 1.603 | 0.257 | -2.313 |
| CSUI_000277 | cyclophilin precursor | protein folding | 0.427 | 1.200 | -0.696 | -3.057 |
| CSUI_004251 | Heat shock protein | protein folding | 1.054 | 2.056 | 0.770 | -1.758 |
| CSUI_000845 | peptidyl-prolyl cis-trans isomerase-like | protein folding | 0.298 | 0.769 | -0.127 | -0.663 |
| CSUI_004971 | t-complex protein 1 subunit alpha | protein folding | 0.657 | 1.011 | 1.267 | 0.373 |
| CSUI_004538 | T-complex protein 1 subunit delta | protein folding | 0.471 | 1.093 | -0.554 | -1.883 |
| CSUI_011238 | T-complex protein 1 subunit eta | protein folding | 0.346 | 1.296 | -0.656 | -3.136 |
| CSUI_008315 | T-complex protein 1 subunit eta (Fragment) | protein folding | 0.401 | 1.008 | -0.747 | -1.332 |
| CSUI_011171 | T-complex protein 1 subunit eta (Fragment) | protein folding | -0.227 | 0.659 | -1.807 | -2.093 |
| CSUI_002777 | T-complex protein 1 subunit gamma | protein folding | 0.064 | 0.463 | 0.117 | -0.550 |
| CSUI_010991 | T-complex protein 1 subunit zeta (Fragment) | protein folding | 0.655 | 1.318 | -0.372 | -2.835 |
| CSUI_003944 | t-complex protein beta subunit | protein folding | -0.179 | 0.552 | -1.097 | -2.555 |
| CSUI_000948 | T-complex protein epsilon subunit (Tcp-1-epsilon) | protein folding | -0.552 | 0.048 | -1.930 | -4.632 |
| CSUI_003267 | T-complex protein epsilon subunit (Tcp-1-epsilon) (Fragment) | protein folding | 0.718 | 1.375 | -0.243 | -2.223 |
| CSUI_000902 | t-complex protein theta subunit | protein folding | 0.720 | 1.672 | -0.169 | -2.668 |
| CSUI_004916 | tcp-1 chaperonin | protein folding | -0.724 | 0.242 | -2.152 | -3.170 |
| CSUI_000616 | Tcp-1 chaperonin (Fragment) | protein folding | 1.338 | 1.566 | 0.112 | -0.368 |
| CSUI_008781 | Tcp-1 cpn60 chaperonin family | protein folding | 0.550 | 0.969 | -0.092 | -1.334 |
| CSUI_003741 | Chaperone protein | protein processing | 1.418 | 2.224 | 0.834 | -1.064 |
| CSUI_001247 | Chaperone protein | protein processing | 1.219 | 2.191 | 0.328 | -1.557 |
| CSUI_010967 | Chaperone related protein | protein processing | 1.138 | 1.491 | -3.342 | -2.745 |
| CSUI_008421 | hypothetical protein | protein processing | 0.510 | 1.569 | -0.443 | -2.805 |
| CSUI_004190 | Protein disulfide isomerase-related protein (Fragment) | protein processing | 0.856 | 1.671 | 0.194 | -2.288 |
| CSUI_008082 | Protein disulfide isomerase-related protein (Fragment) | protein processing | 1.006 | 1.984 | -0.446 | -3.406 |
| CSUI_002634 | Protein disulfide-isomerase | protein processing | 0.711 | 1.398 | -0.273 | -1.552 |
| CSUI_002988 | Protein disulfide-isomerase | protein processing | 0.664 | 1.407 | -0.446 | -3.100 |
| CSUI_011224 | Protein disulfide-isomerase domain-containing | protein processing | 0.915 | 1.596 | -1.568 | -3.217 |
| CSUI_000956 | ribophorin i protein | protein processing | 0.090 | 0.505 | -1.363 | -3.095 |
| CSUI_004062 | emp nonaspanin domain family protein | protein processing | 0.397 | 1.406 | 0.060 | -1.387 |
| CSUI_000857 | Emp24 gp25l p24 family protein | protein processing | 0.799 | 1.765 | -0.150 | -2.980 |
| CSUI_007204 | Emp24 gp25l p24 family protein | protein processing | 0.893 | 1.715 | 0.127 | -2.696 |
| CSUI_005173 | endomembrane protein 70 subfamily protein | protein processing | 1.686 | 2.551 | 1.113 | -0.927 |
| CSUI_002497 | Er lumen protein retaining receptor | protein processing | 0.754 | 1.669 | 0.064 | -2.480 |
| CSUI_000281 | ER membrane protein complex subunit 1 | protein processing | 1.328 | 2.249 | 0.402 | -3.428 |
| CSUI_002389 | Clathrin light chain | intracellular protein transport | 0.000 | 0.234 | 0.751 | 1.231 |
| CSUI_000529 | Protein transport protein SEC23 | intracellular protein transport | -0.904 | -1.129 | -1.181 | -0.051 |
| CSUI_007154 | Nsp1-like carboxy-terminal region | nucleocytoplasmatic transport | 1.462 | 2.514 | 1.353 | 0.296 |
| CSUI_006132 | Exportin | nucleocytoplasmatic transport | 1.220 | 2.074 | 1.327 | -0.366 |
| CSUI_008552 | Importin subunit alpha | nucleocytoplasmatic transport | 0.663 | 1.074 | 0.354 | -1.244 |
| CSUI_004088 | Importin-beta n-terminal domain-containing protein | nucleocytoplasmatic transport | 1.039 | 1.673 | 0.095 | -1.959 |
| CSUI_005298 | Heat repeat-containing protein | nucleocytoplasmatic transport | -0.502 | -0.032 | 0.012 | 0.785 |
| CSUI_003508 | Nucleoporin fg repeat-containing | nucleocytoplasmatic transport | 0.035 | 0.500 | -0.080 | -0.956 |
| CSUI_003287 | Importin subunit beta | nucleocytoplasmatic transport | 0.191 | 0.968 | -0.248 | -1.190 |
| CSUI_010115 | Heat repeat-containing protein | nucleocytoplasmatic transport | 0.407 | 1.485 | -0.908 | -0.210 |
| CSUI_009023 | GTP-binding nuclear protein | nucleocytoplasmatic transport | 0.560 | 1.246 | -1.089 | -2.791 |
| CSUI_002413 | Heat repeat-containing protein | nucleocytoplasmatic transport | -0.080 | 0.130 | -1.761 | -4.212 |
| CSUI_009444 | Signal recognition particle domain protein (Fragment) | protein export | 1.284 | 2.246 | 1.686 | 0.043 |
| CSUI_003541 | Wd g-beta repeat-containing | protein export | -0.183 | 0.087 | 1.489 | 1.130 |
| CSUI_003911 | Microsomal signal peptidase domain-containing protein | protein export | 0.934 | 2.154 | 0.884 | -1.874 |
| CSUI_001208 | Protein transport protein Sec61 subunit beta | protein export | 1.084 | 1.752 | 0.007 | -2.898 |
| CSUI_001117 | Translocation protein SEC62 | protein export | 0.305 | 0.369 | -0.010 | -0.711 |
| CSUI_001290 | Signal peptidase I | protein export | 0.639 | 1.245 | -0.108 | -2.097 |
| CSUI_003447 | Family sec63 protein | protein export | 0.764 | 1.561 | -0.171 | -4.824 |
| CSUI_002005 | Preprotein subunit | protein export | 0.462 | 1.286 | -0.230 | -2.589 |
| CSUI_001468 | Signal peptide peptidase domain-containing | protein export | 0.713 | 1.489 | -0.515 | -3.185 |
| CSUI_002104 | Signal recognition particle protein srp54 | protein export | 0.227 | 1.850 | -0.554 | -0.991 |
| CSUI_003276 | Signal recognition particle receptor subunit beta | protein export | -0.194 | 1.328 | -0.587 | -2.432 |
| CSUI_003697 | Signal peptide peptidase | protein export | 0.313 | 0.438 | -0.616 | -1.304 |
| CSUI_003053 | Eukaryotic porin protein | protein import into mitochondrial matrix | 1.093 | 2.003 | 0.885 | -1.427 |
| CSUI_007200 | Eukaryotic porin domain-containing | protein import into mitochondrial matrix | 1.282 | 2.299 | 0.629 | -2.778 |
| CSUI_003711 | H(+)-exporting diphosphatase | protein transport | 1.335 | 2.697 | 2.203 | -0.020 |
| CSUI_004999 | Coatomer subunit alpha | protein transport | 1.317 | 2.103 | 1.076 | -2.164 |
| CSUI_007363 | Clathrin heavy | protein transport | 0.518 | 0.608 | -0.035 | -0.441 |
| CSUI_004223 | tim10 ddp family zinc finger superfamily protein | protein transport | 0.799 | 1.011 | -0.321 | -1.275 |
| CSUI_008516 | Beta | protein transport | 0.612 | 1.109 | -0.331 | -2.643 |
| CSUI_002793 | tim10 ddp family zinc finger superfamily protein | protein transport | 0.034 | 1.027 | -0.577 | -2.275 |
| CSUI_002451 | Small gtp-binding protein sar1 | protein transport | 0.338 | 0.901 | -0.587 | -3.417 |
| CSUI_011031 | Clathrin heavy (Fragment) | protein transport | 0.642 | 0.828 | -0.778 | -2.549 |
| CSUI_004061 | clathrin heavy | protein transport | 0.727 | 1.175 | -2.471 | -3.908 |
| CSUI_007021 | coatomer alpha | protein transport | -0.528 | 0.492 | -2.779 | -3.568 |
| CSUI_006358 | arf1-directed gtpase-activating | protein transport-endocitosis | -0.141 | 0.596 | -1.043 | -0.841 |
| CSUI_002854 | Snare associated golgi protein | SNARE complex disassembly | 0.747 | 1.459 | -0.128 | -1.472 |
| CSUI_005543 | n-ethylmaleimide-sensitive fusion protein | SNARE complex disassembly | 0.182 | 1.063 | -2.707 | -2.115 |
| CSUI_007005 | Adenine nucleotide | transmembrane transport | 0.940 | 1.815 | 0.606 | -1.600 |
| CSUI_001948 | Abc transporter transmembrane region domain-containing protein (Fragment) | transmembrane transport | 0.907 | 1.663 | 0.175 | -1.352 |
| CSUI_010281 | Formate nitrite transporter protein (Fragment) | transmembrane transport | 0.723 | 1.951 | -0.155 | -0.847 |
| CSUI_009035 | Abc transporter transmembrane region domain-containing protein | transmembrane transport | -0.066 | 0.453 | -0.871 | -2.010 |
| CSUI_001985 | Abc transporter transmembrane region domain-containing protein (Fragment) | transmembrane transport | 1.024 | 1.482 | -1.158 | -4.421 |
| CSUI_003671 | Rap domain protein | vesicles transport | 2.696 | 2.551 | 2.117 | -0.792 |
| CSUI_006797 | Rab 5 | vesicles transport | 1.091 | 2.004 | 0.604 | -1.411 |
| CSUI_005442 | Rab1 protein | vesicles transport | 0.973 | 1.829 | 0.448 | -1.965 |
| CSUI_003665 | Ras-related protein rab11 | vesicles transport | 0.711 | 1.407 | 0.347 | -1.762 |
| CSUI_000619 | Ras-related protein rab-1a | vesicles transport | 0.571 | 1.087 | -0.322 | -3.031 |
| CSUI_003780 | Ran-specific gtpase-activating | vesicles transport | 0.360 | 0.870 | -0.944 | -3.501 |
| CSUI_008850 | Tetratricopeptide repeat-containing protein | ubiquitin-dependent protein catabolic process | -0.545 | 0.094 | 3.015 | 1.687 |
| CSUI_006340 | Heat shock protein hsp28 | ubiquitin-dependent protein catabolic process | 1.152 | 2.199 | 1.172 | -1.533 |
| CSUI_003056 | Tetratricopeptide repeat protein 11 | ubiquitin-dependent protein catabolic process | 0.658 | 1.690 | 0.735 | 0.236 |
| CSUI_004273 | Ubiquitin | ubiquitin-dependent protein catabolic process | 1.066 | 2.596 | 0.645 | -0.328 |
| CSUI_008016 | Ubiquitin | ubiquitin-dependent protein catabolic process | 1.033 | 1.734 | 0.597 | -1.682 |
| CSUI_011134 | Heat shock protein | ubiquitin-dependent protein catabolic process | 1.010 | 2.054 | 0.477 | -1.558 |
| CSUI_000737 | Heat shock protein 90 | ubiquitin-dependent protein catabolic process | 0.891 | 1.611 | 0.355 | -1.740 |
| CSUI_002247 | Hsp70 interacting protein hip | ubiquitin-dependent protein catabolic process | 0.410 | 1.218 | 0.093 | -1.143 |
| CSUI_003613 | Tetratricopeptide repeat-containing protein | ubiquitin-dependent protein catabolic process | 0.503 | 1.024 | -0.207 | -0.797 |
| CSUI_002018 | Ubiquitin-like protein smt3 | ubiquitin-dependent protein catabolic process | 0.827 | 1.342 | -0.249 | -2.162 |
| CSUI_005579 | Ubiquitin carboxyl-terminal hydrolase uchl3 | ubiquitin-dependent protein catabolic process | 0.507 | 1.505 | -0.318 | -2.495 |
| CSUI_001046 | Heat shock protein | ubiquitin-dependent protein catabolic process | 0.261 | 0.898 | -0.326 | -1.318 |
| CSUI_008165 | Heat shock protein hsp90 (Fragment) | ubiquitin-dependent protein catabolic process | 0.158 | 0.770 | -0.681 | -1.729 |
| CSUI_003939 | Tetratricopeptide repeat-containing protein | ubiquitin-dependent protein catabolic process | 0.310 | 1.093 | -0.739 | -2.505 |
| CSUI_000630 | Tetratricopeptide repeat domain containing protein | ubiquitin-dependent protein catabolic process | 0.432 | 1.071 | -0.936 | -2.722 |
| CSUI_002954 | wd g-beta repeat domain containing protein | ubiquitin-dependent protein catabolic process | 0.018 | 0.500 | -0.949 | -1.154 |
| CSUI_006056 | Heat shock protein hsp70 | ubiquitin-dependent protein catabolic process | 0.114 | 0.503 | -1.061 | -2.973 |
| CSUI_011046 | Heat shock protein 90 | ubiquitin-dependent protein catabolic process | 0.060 | 0.735 | -1.076 | -3.906 |
| CSUI_003605 | Heat shock protein | ubiquitin-dependent protein catabolic process | 0.060 | 0.735 | -1.076 | -3.906 |
| CSUI_004788 | Ubiquitin family protein | ubiquitin-dependent protein catabolic process | -0.503 | 0.234 | -1.373 | -0.499 |
| CSUI_008158 | Chaperonin | ubiquitin-dependent protein catabolic process | -0.145 | 0.209 | -1.509 | -2.039 |
| CSUI_000906 | Family chaperone | ubiquitin-dependent protein catabolic process | 0.233 | 0.870 | -1.701 | -3.334 |
| CSUI_001507 | Heat shock protein | ubiquitin-dependent protein catabolic process | 0.325 | 0.809 | -1.854 | -5.136 |
| CSUI_002322 | Heat shock protein 101 (Fragment) | ubiquitin-dependent protein catabolic process | 0.595 | 1.032 | -1.879 | -4.674 |
| CSUI_000585 | Ubiquitin-activating enzyme e1 family protein | ubiquitin-dependent protein catabolic process | -0.320 | 0.464 | -1.977 | -4.007 |
| CSUI_003656 | Heat shock protein hsp29 | ubiquitin-dependent protein catabolic process | 0.342 | 0.773 | -3.365 | -4.557 |
| CSUI_002930 | Aspartyl protease asp1 | proteolysis | 1.522 | 4.068 | 1.958 | 0.030 |
| CSUI_000983 | M28 family protein | proteolysis | 0.635 | 1.660 | 0.864 | -0.533 |
| CSUI_002879 | Subtilisin sub1 | proteolysis | 0.571 | 2.254 | 0.822 | -1.324 |
| CSUI_000501 | CAAX prenyl protease | proteolysis | 1.107 | 1.924 | 0.643 | -1.691 |
| CSUI_008228 | Serine protease | proteolysis | 0.836 | 2.051 | 0.386 | -1.149 |
| CSUI_002902 | Peptidase m16 inactive domain-containing protein | proteolysis | 0.786 | 1.673 | 0.139 | -3.506 |
| CSUI_007107 | Organelle processing peptidase (Fragment) | proteolysis | 0.686 | 1.564 | 0.130 | -2.479 |
| CSUI_000670 | X-pro dipeptidyl-peptidase (S15 family) | proteolysis | 0.801 | 1.689 | 0.113 | -2.353 |
| CSUI_008041 | Peptidase m16 inactive domain-containing protein | proteolysis | -0.022 | 1.096 | 0.089 | -0.830 |
| CSUI_006587 | Peptidase family c78 protein (Fragment) | proteolysis | 0.881 | 1.738 | -0.099 | -0.338 |
| CSUI_002424 | Peptidase amidohydrolase | proteolysis | 1.002 | 2.227 | -0.128 | -3.243 |
| CSUI_002298 | Leucyl aminopeptidase lap | proteolysis | 0.448 | 1.189 | -0.260 | -2.690 |
| CSUI_004037 | Aminopeptidase | proteolysis | 0.432 | 1.040 | -0.447 | -1.986 |
| CSUI_003579 | Subtilisin-like protease | proteolysis | -0.189 | -0.340 | -0.498 | -1.006 |
| CSUI_006232 | Serpin (Serine proteinase inhibitor) superfamily protein | proteolysis | -0.544 | -0.024 | -1.095 | -1.591 |
| CSUI_005505 | Peptidase family m3 protein | proteolysis | 0.280 | 1.590 | -1.398 | -3.995 |
| CSUI_003154 | Serpin (Serine proteinase inhibitor) superfamily protein | proteolysis | 0.223 | 0.732 | -2.009 | -4.011 |
| CSUI_004012 | Cathepsin b | proteolysis | 1.177 | 2.421 | -2.026 | -2.907 |
| CSUI_002714 | Serine protease | proteolysis | 0.033 | 0.555 | -2.209 | -4.011 |
| CSUI_002385 | Peptidase family m3 protein | proteolysis | 0.055 | 0.794 | -2.482 | -2.761 |
| CSUI_007817 | Serine carboxypeptidase s28 protein | proteolysis | -0.386 | -0.532 | -2.645 | -4.512 |
| CSUI_006231 | Serine protease | proteolysis | -0.803 | -0.388 | -3.607 | -6.707 |
| CSUI_003196 | 26s protease regulatory subunit 6b | proteasome complex | 0.016 | -0.361 | 1.433 | 0.684 |
| CSUI_001934 | Proteasome endopeptidase complex | proteasome complex | 1.468 | 1.807 | 1.335 | -1.022 |
| CSUI_002815 | 26s proteasome regulatory subunit 7 | proteasome complex | 1.093 | 1.745 | 0.427 | -1.548 |
| CSUI_002433 | 26s proteasome non-atpase regulatory subunit | proteasome complex | 0.633 | 1.027 | -0.228 | -1.177 |
| CSUI_011275 | 26s protease regulatory subunit (Fragment) | proteasome complex | -0.723 | -0.935 | -0.286 | 0.214 |
| CSUI_004018 | proteasome regulatory subunit | proteasome complex | 0.850 | 1.384 | -0.312 | -2.143 |
| CSUI_001477 | Proteasome subunit alpha type | proteasome complex | 0.021 | 0.498 | -0.324 | -1.578 |
| CSUI_011282 | Proteasome subunit beta | proteasome complex | 0.422 | 0.661 | -0.360 | -0.968 |
| CSUI_007132 | Proteasome subunit alpha type | proteasome complex | 0.650 | 1.557 | -0.455 | -3.093 |
| CSUI_011402 | 26s protease regulatory subunit 4 | proteasome complex | 0.140 | 0.528 | -0.594 | -0.971 |
| CSUI_007878 | Proteasome subunit alpha type | proteasome complex | 0.440 | 1.372 | -0.711 | -3.133 |
| CSUI_000376 | Proteasome subunit beta | proteasome complex | 0.609 | 1.273 | -0.728 | -2.755 |
| CSUI_001286 | 20s proteasome subunit beta | proteasome complex | 0.799 | 1.812 | -0.731 | -1.993 |
| CSUI_005694 | Proteasome endopeptidase complex | proteasome complex | 0.587 | 1.829 | -0.733 | -1.429 |
| CSUI_008383 | 26s proteasome non-atpase regulatory subunit (Fragment) | proteasome complex | -0.104 | 0.156 | -0.786 | -1.017 |
| CSUI_007305 | Proteasome pci domain-containing | proteasome complex | -0.101 | -0.035 | -0.856 | -1.071 |
| CSUI_001855 | Proteasome endopeptidase complex | proteasome complex | 0.025 | 0.760 | -0.986 | -2.854 |
| CSUI_009544 | Proteasome subunit beta type 7 | proteasome complex | 0.158 | 0.995 | -1.508 | -2.988 |
| CSUI_006286 | Proteasome endopeptidase complex | proteasome complex | -0.187 | 0.412 | -1.541 | -2.933 |
| CSUI_002140 | 26s proteasome subunit | proteasome complex | 0.229 | 0.552 | -1.563 | -3.055 |
| CSUI_009239 | t1 family protein | proteasome complex | 0.264 | 1.081 | -1.748 | -3.701 |
| CSUI_002080 | 26s proteasome subunit p55 | proteasome complex | -0.644 | -0.732 | -2.522 | -1.908 |
| CSUI_008687 | Proteasome endopeptidase complex | proteasome complex | -1.612 | 0.857 | -4.495 | -2.666 |
| CSUI_000103 | Inhibitor of cysteine protease 1 | peptidase activity | 0.597 | 1.438 | -0.484 | -3.788 |
| CSUI_000389 | Inhibitor of cysteine protease 1 | peptidase activity | 0.259 | 1.007 | -2.314 | -2.623 |
| CSUI_004036 | aminopeptidase n | aminopeptidase activity | 0.120 | 0.506 | 1.535 | 0.779 |

**Table S7. Proteins with putative roles in the redox homeostasis.** They are listed along with their annotation number in ToxoDB, protein name, biological function, and abundance (LogFC), in each comparison. Early merozoites (day 7, C7) versus late merozoites (day 8, C8), versus sexual stages (day 10, C10), mature sexual stages (day 12, C12) and oocysts (day 14, C14).

| **ProbeID** | **Protein name** | **molecular function** | **C 7_8** | **C 7_10** | **C 7_12** | **C 7_14** |
| --- | --- | --- | --- | --- | --- | --- |
| CSUI_000414 | Mandelonitrile lyase | oxidation-reduction process | -0.746 | 1.185 | 3.052 | 1.463 |
| CSUI_008814 | Peroxiredoxin prx3 | oxidation-reduction process | 1.086 | 2.354 | 1.594 | -0.975 |
| CSUI_010730 | Thioredoxin reductase | oxidation-reduction process | 1.104 | 1.809 | 0.809 | -1.998 |
| CSUI_004157 | Pyridine nucleotide-disulfide oxidoreductase domain-containing protein (Fragment) | oxidation-reduction process | 0.133 | 1.289 | 0.785 | 0.376 |
| CSUI_002731 | Saccharopine dehydrogenase | oxidation-reduction process | 1.152 | 1.987 | 0.562 | -3.373 |
| CSUI_007033 | Glutathione reductase | oxidation-reduction process | 0.562 | 1.431 | 0.184 | -2.464 |
| CSUI_003634 | Nadp-specific glutamate dehydrogenase | oxidation-reduction process | 0.837 | 1.826 | -0.208 | -3.298 |
| CSUI_006609 | Aldehyde dehydrogenase | oxidation-reduction process | 0.195 | 0.982 | -0.241 | -2.747 |
| CSUI_002843 | Flavodoxin domain-containing protein | oxidation-reduction process | 0.651 | 1.238 | -0.497 | -2.632 |
| CSUI_001721 | Peroxiredoxin | oxidation-reduction process | 0.485 | 1.404 | -0.506 | -2.638 |
| CSUI_000587 | Thioredoxin family trp26 protein | oxidation-reduction process | -0.428 | 0.443 | -0.513 | -0.701 |
| CSUI_003931 | Thioredoxin-like protein | oxidation-reduction process | 0.376 | 1.501 | -0.521 | -3.213 |
| CSUI_002376 | Thioredoxin | oxidation-reduction process | 0.558 | 1.119 | -0.544 | -2.139 |
| CSUI_009486 | Peroxiredoxin 6 | oxidation-reduction process | 0.712 | 1.526 | -0.617 | -3.546 |
| CSUI_005966 | Thioredoxin domain-containing protein | oxidation-reduction process | 0.488 | 1.064 | -0.619 | -3.223 |
| CSUI_010413 | Apicoplast-associated thioredoxin family protein atrx1 | oxidation-reduction process | -0.084 | 1.257 | -0.622 | -2.549 |
| CSUI_003530 | Glutaredoxin domain-containing protein | oxidation-reduction process | -0.210 | 0.727 | -0.637 | -0.272 |
| CSUI_007284 | Gmc oxidoreductase | oxidation-reduction process | -0.086 | 0.687 | -0.846 | -2.565 |
| CSUI_000271 | Thioredoxin peroxidase | oxidation-reduction process | 0.267 | 0.898 | -0.897 | -2.119 |
| CSUI_004280 | Short chain dehydrogenase reductase family protein | oxidation-reduction process | 0.676 | 1.219 | -0.963 | -4.493 |
| CSUI_001589 | Thioredoxin domain-containing protein | oxidation-reduction process | 0.477 | 1.107 | -1.175 | -3.416 |
| CSUI_010527 | Glutamate dehydrogenase | oxidation-reduction process | 0.078 | 0.782 | -1.271 | -2.603 |
| CSUI_004134 | Thioredoxin | oxidation-reduction process | -0.493 | 0.236 | -1.753 | -3.173 |
| CSUI_004346 | FeSOD | oxidation-reduction process-peroxisome | 0.779 | 1.708 | 0.473 | -2.396 |
| CSUI_010557 | Catalase (Fragment) | oxidation-reduction process-peroxisome | 0.768 | 1.506 | -0.658 | -1.286 |
| CSUI_005150 | Superoxide dismutase | oxidation-reduction process-peroxisome | -0.379 | 0.261 | -2.470 | -4.651 |
| CSUI_001050 | Aaa family protein | peroxisome | 0.745 | 1.622 | 0.267 | -1.449 |
| CSUI_001732 | Aaa family protein | peroxisome | 0.449 | 0.683 | -1.888 | -3.455 |
| CSUI_004107 | Aaa family protein | peroxisome | 0.179 | 0.133 | -2.032 | -2.485 |

**Table S8. Proteins linked to sexual stages.** They are listed along with their annotation number in ToxoDB, protein name, biological function, and abundance (LogFC), in each comparison. Early merozoites (day 7, C7) versus late merozoites (day 8, C8), versus sexual stages (day 10, C10), mature sexual stages (day 12, C12) and oocysts (day 14, C14).

| **ProbeID** | **Protein name** | **molecular function** | **C 7_8** | **C 7_10** | **C 7_12** | **C 7_14** |
| --- | --- | --- | --- | --- | --- | --- |
| CSUI_000048 | Morn repeat-containing protein | microgametes | 2.469 | 3.378 | 3.858 | 1.490 |
| CSUI_011133 | Membrane occupation and recognition nexus protein morn1 | microgametes | 0.171 | 0.975 | 1.349 | 0.641 |
| CSUI_011036 | Morn repeat protein | microgametes | 1.450 | 2.626 | 1.339 | 0.156 |
| CSUI_007024 | Msp (Major sperm protein) domain-containing protein | microgametes | 0.919 | 1.598 | 0.214 | -2.146 |
| CSUI_009771 | Tubulin beta chain | microtubule-based process | 0.732 | 1.639 | 2.939 | 2.257 |
| CSUI_010140 | Microtubule-binding protein | microtubule-based process | 1.351 | 1.302 | 2.326 | -0.030 |
| CSUI_008696 | Tubulin alpha chain | microtubule-based process | 0.989 | 1.818 | 1.508 | -0.100 |
| CSUI_006267 | Tubulin beta chain | microtubule-based process | 0.662 | 1.727 | 1.362 | -0.476 |
| CSUI_006169 | Tubulin beta chain | microtubule-based process | 1.245 | 2.267 | 1.243 | -0.531 |
| CSUI_003705 | Alveolin domain containing intermediate filament imc12 | oocyst | 4.308 | 7.338 | 6.839 | 3.160 |
| CSUI_003616 | Microneme protein | oocyst | 1.170 | 2.650 | 4.717 | 1.978 |
| CSUI_000999 | Alveolin domain containing intermediate filament imc7 | oocyst | 2.403 | 4.825 | 4.496 | 1.464 |
| CSUI_008977 | Pan domain-containing protein | oocyst | -1.735 | 2.527 | 4.234 | 2.527 |
| CSUI_006641 | Pan domain-containing protein | oocyst | -1.396 | 2.613 | 4.200 | 2.498 |
| CSUI_001473 | TyRP_hypothetical protein | oocyst | -1.525 | 3.393 | 4.031 | 0.869 |
| CSUI_010687 | Pan domain-containing protein | oocyst | 0.420 | 2.217 | 3.432 | 1.766 |
| CSUI_006179 | hypothetical protein-sag related | oocyst | -0.153 | 1.499 | 3.342 | 1.390 |
| CSUI_004248 | Sag-related sequence srs26i | oocyst | -0.119 | 3.367 | 3.288 | 0.877 |
| CSUI_009196 | Toxoplasma gondii family d protein | oocyst | 0.114 | 1.522 | 3.222 | 0.711 |
| CSUI_007432 | Pan domain-containing protein | oocyst | 0.000 | 1.555 | 2.719 | 0.545 |
| CSUI_003908 | Toxoplasma gondii family d protein | oocyst | 0.527 | 1.437 | 2.593 | 0.961 |
| CSUI_010652 | Alveolin domain containing intermediate filament imc7 | oocyst | -0.574 | -0.187 | 1.365 | 1.495 |
| CSUI_008449 | Toxoplasma gondii family a protein | oocyst | -0.721 | 0.218 | 1.287 | 0.622 |
| CSUI_005717 | Imc sub-compartment protein isp1 | oocyst | 0.279 | 1.040 | 1.269 | 1.245 |
| CSUI_002476 | Outer omp85 family protein | oocyst | 0.961 | 1.304 | 0.725 | -2.205 |
| CSUI_010158 | Toxoplasma gondii family a protein | oocyst | 0.586 | 0.940 | -0.879 | -2.475 |

**Table S9. Cell invasion related proteins.** They are listed along with their annotation number in ToxoDB, protein name, biological function, and abundance (LogFC), in each comparison. Early merozoites (day 7, C7) versus late merozoites (day 8, C8), versus sexual stages (day 10, C10), mature sexual stages (day 12, C12) and oocysts (day 14, C14).

| **ProbeID** | **Protein name** | **Function** | **C 7_8** | **C7_10** | **C 7_12** | **C 7_14** |
| --- | --- | --- | --- | --- | --- | --- |
| CSUI_006251 | Gap40 protein | gliding motility | 0.567 | 1.241 | -1.413 | -5.069 |
| CSUI_000298 | Glideosome-associated protein with multiple-membrane spans gapm3 | gliding motility | 0.612 | 0.920 | -1.756 | -4.003 |
| CSUI_007151 | Gap45 protein | gliding motility | 0.300 | 1.084 | -2.108 | -6.547 |
| CSUI_001007 | Glideosome-associated protein with multiple-membrane spans gapm2b | gliding motility | -0.007 | 0.948 | -2.319 | -2.595 |
| CSUI_001743 | Inner membrane protein | IMC complex | 0.488 | 1.380 | 0.652 | -0.203 |
| CSUI_001391 | Membrane skeletal protein | IMC complex | 0.774 | 1.483 | -0.757 | -2.703 |
| CSUI_001147 | Photosensitized ina-labeled protein phil1 | IMC complex | 0.993 | 1.571 | -0.933 | -5.511 |
| CSUI_009009 | Inner membrane complex protein | IMC complex | 0.475 | 1.373 | -1.148 | -3.219 |
| CSUI_004345 | Inner membrane complex protein 22 | IMC complex | 0.932 | 1.638 | -1.175 | -2.368 |
| CSUI_001390 | Membrane skeletal protein | IMC complex | 0.662 | 1.411 | -1.335 | -4.843 |
| CSUI_003486 | Alveolin domain containing intermediate filament imc6 | IMC complex | 0.493 | 1.223 | -1.465 | -3.831 |
| CSUI_001537 | Alveolin domain containing intermediate filament imc10 | IMC complex | 0.414 | 1.110 | -1.514 | -5.345 |
| CSUI_002278 | Inner membrane complex protein 24 | IMC complex | 0.634 | 1.377 | -1.830 | -3.983 |
| CSUI_000302 | Inner membrane complex protein 20 | IMC complex | 0.782 | 1.576 | -1.836 | -2.348 |
| CSUI_001891 | Imc sub-compartment protein isp2 | IMC complex | 0.466 | 1.022 | -2.078 | -4.596 |
| CSUI_003864 | Alveolin domain containing intermediate filament imc12 | IMC complex | -0.432 | 0.054 | -2.172 | -2.901 |
| CSUI_005485 | Imc sub-compartment protein isp3 | IMC complex | 0.324 | 0.881 | -2.718 | -3.224 |
| CSUI_009555 | Alveolin domain containing intermediate filament imc14 | IMC complex | 0.405 | 0.584 | -3.944 | -4.419 |
| CSUI_002095 | Inner membrane complex protein imc2a | IMC complex | -0.380 | -0.309 | -4.593 | -4.525 |
| CSUI_004172 | Wd g-beta repeat-containing protein | microtubule | 1.555 | 2.285 | 0.849 | 0.229 |
| CSUI_003273 | Myosin heavy chain | Microtubule | 0.477 | 1.141 | -0.306 | -2.175 |
| CSUI_010344 | Myosin f | Microtubule | 0.502 | 1.227 | -0.553 | -1.986 |
| CSUI_004717 | Dynein light chain | microtubule | 0.369 | 0.237 | -0.584 | -1.621 |
| CSUI_004127 | Myosin a | Microtubule | 0.535 | 0.986 | -0.951 | -2.340 |
| CSUI_010541 | Myosin d | Microtubule | 0.840 | 0.928 | -1.311 | -3.242 |
| CSUI_007601 | Myosin f | Microtubule | 0.190 | 0.316 | -1.422 | -0.920 |
| CSUI_005337 | Myosin light chain | Microtubule | 0.396 | 0.792 | -1.856 | -3.435 |
| CSUI_003685 | Dynein light intermediate chain | microtubule | -0.067 | 0.587 | -1.905 | -2.638 |
| CSUI_000063 | Myosin d | Microtubule | 0.630 | 0.768 | -2.226 | -3.383 |
| CSUI_007002 | Centrin 2 | microtubule | -0.268 | 0.477 | -0.427 | -0.766 |
| CSUI_001393 | tubulin alpha chain | microtubule-based process | -0.477 | -1.038 | -1.556 | -0.391 |
| CSUI_003198 | p25-alpha family protein | microtubule-based process | -0.780 | -1.108 | -2.140 | -2.079 |
| CSUI_011427 | myosin f | microtubule-based process | -0.573 | -0.015 | 0.307 | 0.645 |
| CSUI_001098 | Apical membrane antigen 1 domain-containing protein | moving junction | 0.663 | 1.505 | -0.323 | -1.523 |
| CSUI_003734 | apical cap protein 1 | moving junction | 0.278 | 0.817 | -1.361 | -2.451 |
| CSUI_001734 | Protein phosphatase 2c domain-containing protein | protein dephosphorylation | 0.942 | 1.051 | 0.674 | -0.823 |
| CSUI_009273 | Protein phosphatase 2c domain-containing protein | protein dephosphorylation | 0.572 | 1.027 | -0.253 | -1.857 |
| CSUI_008364 | Protein phosphatase 2c domain-containing protein | protein dephosphorylation | 0.482 | 0.870 | -2.651 | -3.586 |
| CSUI_001629 | Agc kinase | protein phosphorylation | 0.554 | 0.813 | -0.166 | -1.467 |
| CSUI_010907 | Calcium-dependent protein kinase cdpk3 | protein phosphorylation | 0.720 | 1.101 | -1.201 | -2.913 |
| CSUI_001769 | Camp-dependent protein kinase regulatory subunit | protein phosphorylation | 0.115 | 0.544 | -2.392 | -4.898 |
| CSUI_000151 | Camk cdpk protein kinase | protein phosphorylation | -0.046 | 0.291 | -2.740 | -4.404 |
| CSUI_007612 | Calcium-dependent protein kinase cdpk3 | protein phosphorylation | 0.778 | 0.872 | -4.657 | -5.297 |
| CSUI_005822 | Diacylglycerol kinase (ATP) | signal transduction | 0.081 | 0.155 | -1.143 | -0.133 |
| CSUI_009634 | hypothetical protein | Adhesion/Invasion | 0.717 | 1.386 | -0.482 | -2.620 |
| CSUI_000399 | Platelet-binding protein | adhesion/Invasion | -0.008 | 0.369 | -1.664 | -2.728 |
| CSUI_000919 | Cytoadherence-linked asexual protein | adhesion/Invasion | 0.054 | 0.069 | -2.197 | -2.494 |
| CSUI_003298 | Dense granule protein gra9 | Biogenesis and maturation of the PV | 0.635 | 0.906 | -1.156 | -3.326 |
| CSUI_000780 | Dense-granule antigen dg32 | Biogenesis and maturation of the PV | 0.479 | 0.526 | -1.736 | -4.796 |
| CSUI_006321 | Microneme protein mic4 | Host cell-attachment/Invasion | 1.778 | 1.865 | 0.712 | -3.643 |
| CSUI_010823 | Microneme protein mic4 | Host cell-attachment/Invasion | 0.379 | 0.905 | -0.739 | -2.323 |
| CSUI_006265 | Microneme protein mic4 | Host cell-attachment/Invasion | 1.416 | 1.397 | -1.424 | -4.474 |
| CSUI_008960 | Mic2-associated protein m2ap | Host cell-attachment/Invasion | 0.149 | -0.511 | -2.078 | -2.141 |
| CSUI_001458 | Microneme protein 13 | Host cell-attachment/Invasion | 1.534 | 1.459 | -2.111 | -2.462 |
| CSUI_003520 | Microneme protein | Host cell-attachment/Invasion | 0.430 | 1.060 | -2.263 | -4.848 |
| CSUI_000791 | Microneme protein mic12 | Host cell-attachment/Invasion | 0.466 | 0.392 | -2.266 | -2.516 |
| CSUI_001209 | Microneme protein 13 | Host cell-attachment/Invasion | -0.383 | -0.390 | -2.370 | -1.987 |
| CSUI_000673 | Microneme protein mic6 | Host cell-attachment/Invasion | 0.660 | 0.825 | -2.397 | -2.745 |
| CSUI_010224 | Pan domain-containing protein | Host cell-attachment/Invasion | 0.605 | 0.344 | -2.975 | -3.995 |
| CSUI_002748 | Microneme protein | Host cell-attachment/Invasion | 0.203 | 0.315 | -3.208 | -3.483 |
| CSUI_003617 | Microneme protein 13 | Host cell-attachment/Invasion | 0.147 | 0.200 | -4.339 | -4.447 |
| CSUI_009127 | Pb-reticulocyte-binding protein | Invasion/Virulence | 1.402 | 2.210 | 0.662 | -3.236 |
| CSUI_003834 | Rhoptry kinase family protein rop28 | Invasion/Virulence | 0.353 | 0.397 | -0.894 | -1.226 |
| CSUI_005981 | Rhoptry kinase family protein rop11 (Incomplete catalytic triad) | Invasion/Virulence | -0.849 | -0.643 | -1.264 | -1.787 |
| CSUI_005876 | Rhoptry neck protein ron10 | Invasion/Virulence | 0.220 | 0.362 | -1.314 | -2.272 |
| CSUI_008980 | Rhoptry kinase family protein rop32 | Invasion/Virulence | -1.061 | -0.682 | -1.629 | -2.024 |
| CSUI_007149 | Rhoptry neck protein ron3 | Invasion/Virulence | 0.386 | 0.703 | -1.666 | -3.575 |
| CSUI_005741 | Rhoptry kinase family protein rop11 (Incomplete catalytic triad) | Invasion/Virulence | 0.410 | 0.380 | -1.853 | -4.447 |
| CSUI_004042 | Rhoptry neck protein ron4 | Invasion/Virulence | 0.483 | 0.601 | -2.211 | -4.637 |
| CSUI_007069 | Rhoptry neck protein ron8 | Invasion/Virulence | 0.112 | -0.256 | -2.322 | -1.171 |
| CSUI_002983 | Rhoptry protein rop12 | Invasion/Virulence | 0.051 | 0.192 | -2.356 | -4.139 |
| CSUI_009576 | Rhoptry protein rop17 | Invasion/Virulence | 0.630 | 0.847 | -2.584 | -3.492 |
| CSUI_004303 | Rhoptry kinase family protein rop35 | Invasion/Virulence | -1.183 | -0.590 | -2.682 | -5.211 |
| CSUI_007315 | rhoptry neck protein | Invasion/Virulence | 0.007 | 0.032 | -2.761 | -3.390 |
| CSUI_007762 | Rhoptry kinase family protein rop32 | Invasion/Virulence | 0.059 | -0.011 | -2.905 | -4.002 |
| CSUI_004221 | Rhoptry kinase family protein rop28 | Invasion/Virulence | 0.170 | 0.040 | -3.202 | -4.094 |
| CSUI_004499 | Rhoptry kinase family protein rop32 | Invasion/Virulence | 0.690 | 0.851 | -3.317 | -4.183 |
| CSUI_004598 | Rhoptry kinase family protein rop28 | Invasion/Virulence | 0.065 | -0.707 | -5.774 | -6.262 |
| CSUI_000216 | Pan domain-containing protein | proteolysis | -2.401 | -2.388 | -1.867 | 0.362 |
| CSUI_001774 | Pan domain-containing protein | proteolysis | -0.194 | -0.083 | -2.795 | -3.971 |
| CSUI_003743 | Sag-related sequence srs33 | Adhesion/Invasion | 2.299 | 3.039 | 0.990 | -1.941 |
| CSUI_007676 | Sag-related sequence srs53c | Adhesion/Invasion | 1.888 | 2.948 | 0.618 | -2.356 |
| CSUI_001005 | SAG domain-containing protein | Adhesion/Invasion | 1.590 | 2.820 | 0.583 | -1.517 |
| CSUI_003351 | Srs domain-containing protein | Adhesion/Invasion | 2.985 | 3.648 | 0.565 | -1.786 |
| CSUI_002410 | Srs domain-containing protein | Adhesion/Invasion | 1.545 | 2.525 | 0.492 | -2.108 |
| CSUI_005991 | SAG domain-containing protein | Adhesion/Invasion | 1.159 | 1.707 | 0.431 | -2.238 |
| CSUI_010845 | Srs domain-containing protein | Adhesion/Invasion | 1.237 | 1.563 | 0.330 | -1.266 |
| CSUI_009427 | Srs domain-containing protein | Adhesion/Invasion | 1.494 | 2.669 | 0.296 | -2.385 |
| CSUI_003744 | Sag-related sequence srs25 | Adhesion/Invasion | 0.680 | 1.195 | 0.272 | -0.968 |
| CSUI_011375 | Srs domain-containing protein | Adhesion/Invasion | 1.237 | 1.921 | 0.013 | -3.202 |
| CSUI_009618 | Sag-related sequence srs22c | Adhesion/Invasion | 1.100 | 1.421 | 0.004 | -1.626 |
| CSUI_002409 | Srs domain-containing protein | Adhesion/Invasion | 1.207 | 2.257 | -0.009 | -2.534 |
| CSUI_009057 | Srs domain-containing protein | Adhesion/Invasion | 1.099 | 2.063 | -0.105 | -4.084 |
| CSUI_005990 | srs domain-containing protein | Adhesion/Invasion | 1.805 | 2.513 | -0.128 | -0.442 |
| CSUI_003350 | Srs domain-containing protein | Adhesion/Invasion | 0.834 | 1.368 | -0.260 | -1.782 |
| CSUI_009053 | Srs domain-containing protein | Adhesion/Invasion | 1.064 | 2.135 | -0.335 | -4.127 |
| CSUI_002641 | Sag-related sequence srs53c | Adhesion/Invasion | 0.831 | 1.620 | -0.445 | -0.758 |
| CSUI_000323 | SAG domain-containing protein | Adhesion/Invasion | 1.044 | 1.635 | -0.470 | -3.090 |
| CSUI_009633 | Sag-related sequence srs11 | Adhesion/Invasion | 0.717 | 1.386 | -0.482 | -2.620 |
| CSUI_005472 | Sag-related sequence srs60a | Adhesion/Invasion | 1.015 | 1.484 | -0.503 | -2.896 |
| CSUI_005474 | sag-related sequence srs60a | Adhesion/Invasion | 1.998 | 2.285 | -0.571 | -2.395 |
| CSUI_009058 | Srs domain-containing protein | Adhesion/Invasion | 0.710 | 1.413 | -0.603 | -1.599 |
| CSUI_006891 | Sag-related sequence srs60a | Adhesion/Invasion | 0.998 | 1.807 | -0.609 | -3.060 |
| CSUI_005469 | Srs domain-containing protein | Adhesion/Invasion | 0.825 | 1.806 | -0.665 | -3.100 |
| CSUI_006047 | Srs domain-containing protein | Adhesion/Invasion | 0.866 | 1.422 | -0.672 | -4.291 |
| CSUI_001006 | SAG domain-containing protein | Adhesion/Invasion | 1.156 | 2.051 | -0.693 | -1.184 |
| CSUI_000324 | SAG domain-containing protein | Adhesion/Invasion | 1.432 | 2.058 | -0.705 | -1.248 |
| CSUI_007477 | Sag-related sequence srs53c | Adhesion/Invasion | 0.731 | 1.276 | -0.718 | -2.837 |
| CSUI_008565 | Srs domain-containing protein | Adhesion/Invasion | 0.700 | 1.316 | -0.765 | -4.116 |
| CSUI_001533 | Sag-related sequence srs53f | Adhesion/Invasion | 1.035 | 1.274 | -0.853 | -0.805 |
| CSUI_009059 | Sag-related sequence srs30d | Adhesion/Invasion | 1.013 | 1.603 | -0.899 | -2.900 |
| CSUI_009181 | SAG domain-containing protein | Adhesion/Invasion | 1.067 | 1.611 | -0.948 | -4.837 |
| CSUI_009426 | Srs domain-containing protein | Adhesion/Invasion | 0.642 | 1.491 | -0.983 | -3.001 |
| CSUI_006282 | Srs domain-containing protein | Adhesion/Invasion | 1.009 | 1.858 | -1.068 | -2.615 |
| CSUI_004246 | Sag-related sequence srs26j | Adhesion/Invasion | 0.548 | 1.068 | -1.072 | -3.248 |
| CSUI_008233 | SAG domain-containing protein | Adhesion/Invasion | 0.672 | 1.422 | -1.084 | -2.193 |
| CSUI_009012 | Srs domain-containing protein | Adhesion/Invasion | 1.010 | 1.326 | -1.108 | -5.294 |
| CSUI_005950 | Sag-related sequence srs20c | Adhesion/Invasion | 0.937 | 2.094 | -1.176 | -2.956 |
| CSUI_007678 | Sag-related sequence srs53f | Adhesion/Invasion | 1.020 | 1.289 | -1.247 | -3.667 |
| CSUI_010483 | SAG domain-containing protein | adhesion/Invasion | -0.417 | -0.408 | -1.334 | -0.374 |
| CSUI_010479 | Sag-related sequence srs53f | Adhesion/Invasion | 1.406 | 1.621 | -1.380 | -3.629 |
| CSUI_007474 | Sag-related sequence srs53f | Adhesion/Invasion | 0.881 | 1.547 | -1.477 | -4.032 |
| CSUI_004444 | Sag-related sequence srs26i | Adhesion/Invasion | 0.636 | 1.219 | -1.477 | -3.918 |
| CSUI_011516 | Sag-related sequence srs53f | Adhesion/Invasion | 0.807 | 1.548 | -1.481 | -3.049 |
| CSUI_010484 | Sag-related sequence srs53f | Adhesion/Invasion | 0.831 | 1.129 | -1.598 | -3.060 |
| CSUI_003934 | SAG domain-containing protein | Adhesion/Invasion | 0.930 | 0.863 | -1.615 | -3.539 |
| CSUI_009424 | Srs domain-containing protein | Adhesion/Invasion | 0.834 | 1.096 | -1.627 | -2.441 |
| CSUI_008686 | Srs domain-containing protein | Adhesion/Invasion | 0.869 | 1.221 | -1.645 | -3.521 |
| CSUI_009947 | Srs domain-containing protein | Adhesion/Invasion | 0.745 | 0.989 | -1.681 | -4.612 |
| CSUI_001833 | Surface antigen | Adhesion/Invasion | 0.317 | 0.916 | -1.686 | -3.421 |
| CSUI_008558 | SAG domain-containing protein | Adhesion/Invasion | 0.935 | 1.416 | -1.729 | -4.772 |
| CSUI_003788 | Srs domain-containing protein | Adhesion/Invasion | 1.578 | 2.120 | -1.777 | -2.546 |
| CSUI_010847 | Srs domain-containing protein | Adhesion/Invasion | 0.631 | 0.850 | -1.840 | -3.997 |
| CSUI_005473 | Sag-related sequence srs28 | Adhesion/Invasion | 1.287 | 1.683 | -1.886 | -3.044 |
| CSUI_006281 | Srs domain-containing protein | Adhesion/Invasion | 0.266 | 0.990 | -2.055 | -3.647 |
| CSUI_002407 | Srs domain-containing protein | Adhesion/Invasion | 0.719 | 1.294 | -2.056 | -4.772 |
| CSUI_003092 | Sag-related sequence srs17a | Adhesion/Invasion | 1.064 | 1.490 | -2.079 | -4.785 |
| CSUI_009011 | Srs domain-containing protein | Adhesion/Invasion | 0.824 | 1.003 | -2.119 | -5.316 |
| CSUI_008559 | Srs domain-containing protein | Adhesion/Invasion | 0.897 | 1.275 | -2.131 | -3.176 |
| CSUI_000034 | Sag-related sequence srs53c | Adhesion/Invasion | 0.602 | 1.121 | -2.181 | -4.155 |
| CSUI_003013 | SAG domain-containing protein | Adhesion/Invasion | 0.907 | 1.114 | -2.187 | -4.725 |
| CSUI_000322 | Sag-related sequence srs13 | Adhesion/Invasion | 0.133 | 0.750 | -2.243 | -4.668 |
| CSUI_009013 | Srs domain-containing protein | Adhesion/Invasion | -1.142 | -0.977 | -2.310 | -1.715 |
| CSUI_003681 | Surface antigen 2 | adhesion/Invasion | -0.961 | -0.170 | -2.312 | -2.049 |
| CSUI_010578 | Sag-related sequence srs57 | adhesion/Invasion | -0.052 | 0.259 | -2.343 | -1.860 |
| CSUI_010846 | Srs domain-containing protein | Adhesion/Invasion | 0.412 | 0.625 | -2.349 | -5.263 |
| CSUI_007605 | SAG domain-containing protein | Adhesion/Invasion | 1.418 | 1.454 | -2.359 | -1.942 |
| CSUI_010529 | Srs domain-containing protein | Adhesion/Invasion | 0.862 | 1.073 | -2.607 | -4.351 |
| CSUI_010203 | Srs domain-containing protein | Adhesion/Invasion | 0.390 | 0.681 | -2.837 | -4.646 |
| CSUI_005568 | Sag-related sequence srs17b | Adhesion/Invasion | 0.419 | 0.090 | -3.672 | -4.895 |
| CSUI_003818 | Srs domain-containing protein | Adhesion/Invasion | 0.200 | -0.806 | -3.700 | -3.883 |
