# Supplementary material for: Unravelling the sexual developmental biology of Cystoisospora suis, a model for comparative coccidian parasite studies
Source: Front Cell Infect Microbiol. 2023 Oct 25;13:1271731. doi: 10.3389/fcimb.2023.1271731 (PMC10635411; doi:10.3389/fcimb.2023.1271731)
Supplement: Supplementary file 7 [file DataSheet_1.docx]

**Supplemental Materials and Methods**

**Validation of the filtering criteria and method**

Our chosen filtering criterion was a compromise between information for imputation and keeping as many proteins as possible for hypothesis testing across all developmental stages especially for downregulated proteins in the latest developmental stage, and to base further downstream analysis on the largest possible set of proteins.

The missing data was imputed with kNN methodology of 10 nearest neighbors based on Euclidean distances before hypothesis testing.

We reason that the chosen filtering criteria and kNN method of imputation should not result in an excess of false positively identified, especially downregulated, proteins but rather a possible loss of power and a possible downwards bias of effect sizes for contrasts including day 14 in particular. Nevertheless, a large number of downregulated proteins were identified in contrasts involving timepoint 14.

To assess whether our filtering and imputation strategy affected our results and conclusions drawn we also ran the analysis on the set of 654 proteins that were measured in all 40 samples therefore voiding the imputation step. Supplemental Figure S4 shows detected patterns of expression changes for the 634 proteins that were statistically significant at global 5% FDR and a minimum log2 Fold Change *>=|1|* in at least one of the 10 contrasts tested for the 654 proteins that were quantified in all 40 samples (634/654=96.9% of proteins).

The clusters with the largest number of proteins that exhibit a shared expression change pattern over time are the same among the non-imputed proteins (Figures S4) and the data that includes imputed proteins shown in Figure 1B. We are therefore confident that our chosen filtering and imputation strategy did not introduce biases that would impact our conclusions, but our conclusions were rather made based on a larger set of proteins.

Imputation with zero or drawing from Gaussian distributions centered around a minimal value (MinProb, where we tried different percentiles for the minimal value) all led to severe variance inhomogeneity in the data, therefore violating assumption for the linear mixed models required for hypothesis testing, given the covariance structure in our data.
